# Mechanisms for mTORC1 activation and synergistic induction of apoptosis by ruxolitinib and BH3 mimetics or autophagy inhibitors in JAK2-V617F-expressing leukemic cells including newly established PVTL-2

**DOI:** 10.18632/oncotarget.25515

**Published:** 2018-06-01

**Authors:** Shinya Ishida, Hiroki Akiyama, Yoshihiro Umezawa, Keigo Okada, Ayako Nogami, Gaku Oshikawa, Toshikage Nagao, Osamu Miura

**Affiliations:** ^1^ Department of Hematology, Graduate School of Medical and Dental Sciences, Tokyo Medical and Dental University, Tokyo, Japan

**Keywords:** JAK2-V617F, BH3 mimetic, MPN, apoptosis, mTOR

## Abstract

The activated JAK2-V617F mutant is very frequently found in myeloproliferative neoplasms (MPNs), and its inhibitor ruxolitinib has been in clinical use, albeit with limited efficacies. Here, we examine the signaling mechanisms from JAK2-V617F and responses to ruxolitinib in JAK2-V617F-positive leukemic cell lines, including PVTL-2, newly established from a patient with post-MPN secondary acute myeloid leukemia, and the widely used model cell line HEL. We have found that ruxolitinib downregulated the mTORC1/S6K/4EBP1 pathway at least partly through inhibition of the STAT5/Pim-2 pathway with concomitant downregulation of c-Myc, MCL-1, and BCL-xL as well as induction of autophagy in these cells. Ruxolitinib very efficiently inhibited proliferation but only modestly induced apoptosis. However, inhibition of BCL-xL/BCL-2 by the BH3 mimetics ABT-737 and navitoclax or BCL-xL by A-1331852 induced caspase-dependent apoptosis involving activation of Bak and Bax synergistically with ruxolitinib in HEL cells. On the other hand, the putative pan-BH3 mimetic obatoclax as well as chloroquine and bafilomycin A1 inhibited autophagy at its late stage and induced apoptosis in PVTL-2 cells synergistically with ruxolitinib. The present study suggests that autophagy as well as the anti-apoptotic BCL-2 family members, regulated at least partly by the mTORC1 pathway downstream of STAT5/Pim-2, protects JAK2-V617F-positive leukemic cells from ruxolitinib-induced apoptosis depending on cell types and may contribute to development of new strategies against JAK2-V617F-positive neoplasms.

## INTRODUCTION

The Janus kinase (JAK) family of cytoplasmic tyrosine kinases, comprised of JAK1, JAK2, JAK3, and TYK2, couples with cytokine receptors upon ligand binding and plays essential roles in transduction of intracellular signaling from these receptors lacking the tyrosine kinase domain [1]. Among these kinases, JAK2 plays a crucial role in regulation of proliferation and apoptosis of hematopoietic cells by activating various signaling pathways including the STAT5, Ras/Raf-1/MEK/Erk, and PI3K/Akt/mTOR pathways [2]. The somatic mutation JAK2-V617F is frequently observed in BCR/ABL1-negative myeloproliferative neoplasms (MPNs): 92% in polycythemia vera (PV), 55% in essential thrombocythemia (ET), and 50% in primary myelofibrosis (PMF) [3]. Some cases of PMF or PV, and less frequently those of ET, progress and transform into secondary AML (post-MPN sAML) with its frequency increased up to 20% in patients treated with chemotherapy. However, the significance of JAK2-V617F in the evolution of MPNs remains unknown, because about 40% of the cases lose JAK2-V617F after transformation to sAML [3]. JAK2-V617F is activated constitutively and stimulates the various signaling pathways downstream of JAK2 in cytokine-stimulated cells, thus leading to cytokine-independent cell survival and proliferation when expressed in cytokine-dependent hematopoietic cell lines and causing phenotypes similar to PV in various murine models [1, 2, 4]. Various studies on JAK2-mediated signaling and leukemogenesis have also utilized several JAK2-V617F-positive cell lines derived from patients with post-MPN sAML [5], including the PVTL-1 cell line we previously established from a patient with AML evolving from PV [6]. A number of JAK inhibitors have been developed and under clinical trials for various neoplastic and autoimmune disorders [4]. However, only the JAK1/JAK2 inhibitor ruxolitinib has been approved for clinical use against MPNs, including PMF and PV, with only limited efficacies, which may be partly because of their inherent myelosuppressive effects due to inhibition of normal JAK2 and inability to reduce JAK2-positive neoplastic cells significantly. Furthermore, ruxolitinib has shown only transient and limited effects against post-MPN sAML, which bears the uniformly dismal prognosis with median survival of less than 6 months [7, 8]. In this regard, it has been reported that JAK2-V617F-positive cell lines readily gain resistance to JAK inhibitors after a long-term exposure to gradually increasing concentrations of these inhibitors [9–12]. Thus, development of newer therapeutic strategies for MPNs and, particularly, post-MPN sAML is urgently needed.

The mTOR signaling pathway is mainly activated downstream of the PI3K/Akt pathway in a variety of circumstances and plays key roles in regulation of cell proliferation, apoptosis, autophagy, and metabolism of a variety of cells [13, 14]. Of the two multi-protein complexes formed by the serine/threonine kinase mTOR, mTORC1 plays a critical role in regulation of cap-dependent translation of mRNAs through phosphorylation of 4EBP1 as well as inhibition of autophagy. The phosphorylation of 4EBP1 leads to its dissociation from the mRNA m^7^-GTP cap-binding protein eIF4E to allow its interaction with the scaffolding protein eIF4G to initiate the formation of the translation-initiating complex eIF4F. This complex is required for the translation of mRNAs containing long 5’-UTRs, which are highly structured and have a high G+C content, such as those for c-Myc, MCL-1 and cyclin D1. Although the mTORC1 activity has been reported to be upregulated in primary MPN cells with its inhibition leading to suppression of cell proliferation [6, 15–17], its activation mechanisms have not precisely been elucidated with its possible relationship with the STAT5 pathway activated by JAK2-V617F unknown.

Apoptosis contributes significantly to the clinical effects of various chemotherapies and molecularly targeted therapies for hematological malignancies as well as solid tumors [18]. The intrinsic or mitochondrial apoptotic pathway is tightly regulated by the BCL-2 family of proteins, which is classified into three subgroups. The anti-apoptotic or pro-survival BCL-2 proteins, such as BCL-2, BCL-xL, and MCL-1, bind and inhibit the action of pro-apoptotic proteins. The pro-apoptotic members include the multi-BH-domain effectors Bax and Bak as well as the BH3-only members, such as Bim, Bad, and Bid, which may directly bind and activate Bax/Bak or bind and neutralize the anti-apoptotic BCL-2 family members. Upon activation, Bak and Bax undergo conformational changes, mitochondrial translocation, and oligomerization leading to mitochondrial outer membrane permeabilization, which results in the release of cytochrome c and the downstream activation of the caspase cascade triggering cell death. Thus, the functional balance between pro-and anti-apoptotic members forms a complex interaction network regulating cell fate. BH3 mimetics are small-molecule inhibitors targeting specific members of the anti-apoptotic BCL-2 family members to disrupt interaction with the pro-apoptotic members. Venetoclax specifically targeting BCL-2 has been approved for treatment of chronic lymphocytic leukemia, and several other BH3 mimetics, such as navitoclax targeting BCL-2, BCL-xL, and BCL-w, are currently under clinical trials for several hematological malignancies [18].

Many stresses sequentially elicit autophagy and apoptosis within the same cell [19, 20]. In many cases, autophagy constitutes a strategy to adapt to and cope with stress to prevent apoptosis, generally through nutrient recycling and the clearance of damaged organelles or proteins. On the other hand, in response to growth factor signaling, mTORC1 directly blocks the induction of autophagy by promoting inhibitory phosphorylation of ULK1 [21]. As the mTOR pathway is often activated aberrantly in AML, its inhibitors have been shown to induce autophagy, as judged by increased expression of LC3-II, appearance of punctate GFP-LC3-positive autophagosomes, and decreased expression of p62/SQSTM1 [22, 23]. Recent studies have revealed that autophagy is a major contributor to chemotherapy resistance in AML, and autophagy inhibition strategies mainly using the only currently available clinical inhibitor hydroxychloroquine have been under preclinical studies to enhance efficiencies of chemotherapies and several molecularly targeted therapies [20]. However, the possible involvement and significance of autophagy in response to ruxolitinib in JAK2-V617F-positive cells have mainly remained unknown.

In the present study, we have established a new JAK2-V617F-expressing leukemic cell line, PVTL-2, derived from the same patient with post-PV sAML from whom we previously established PVTL-1 [6]. We have then analyzed intracellular signaling mechanisms from JAK2-V617F and effects of ruxolitinib and other JAK inhibitors on these signaling events and on cell proliferation, apoptosis, and autophagy in PVTL-2 in comparison with the commonly used JAK2-V617F-positive cell line HEL. We have shown involvement of the STAT5/Pim-2 pathway in enhancement of the mTORC1 pathway in these cells and revealed synergistic effects of BH3 mimetics or autophagy inhibitors with ruxolitinib to induce apoptosis mainly in HEL or PVTL-2 cells, respectively. These results may contribute to studies on development of new strategies against JAK2-V617F-positive neoplasms.

## RESULTS

### Morphological, immunophenotypic, cytogenetic, and genetic characterization of PVTL-2 cells

**Figure 1:**
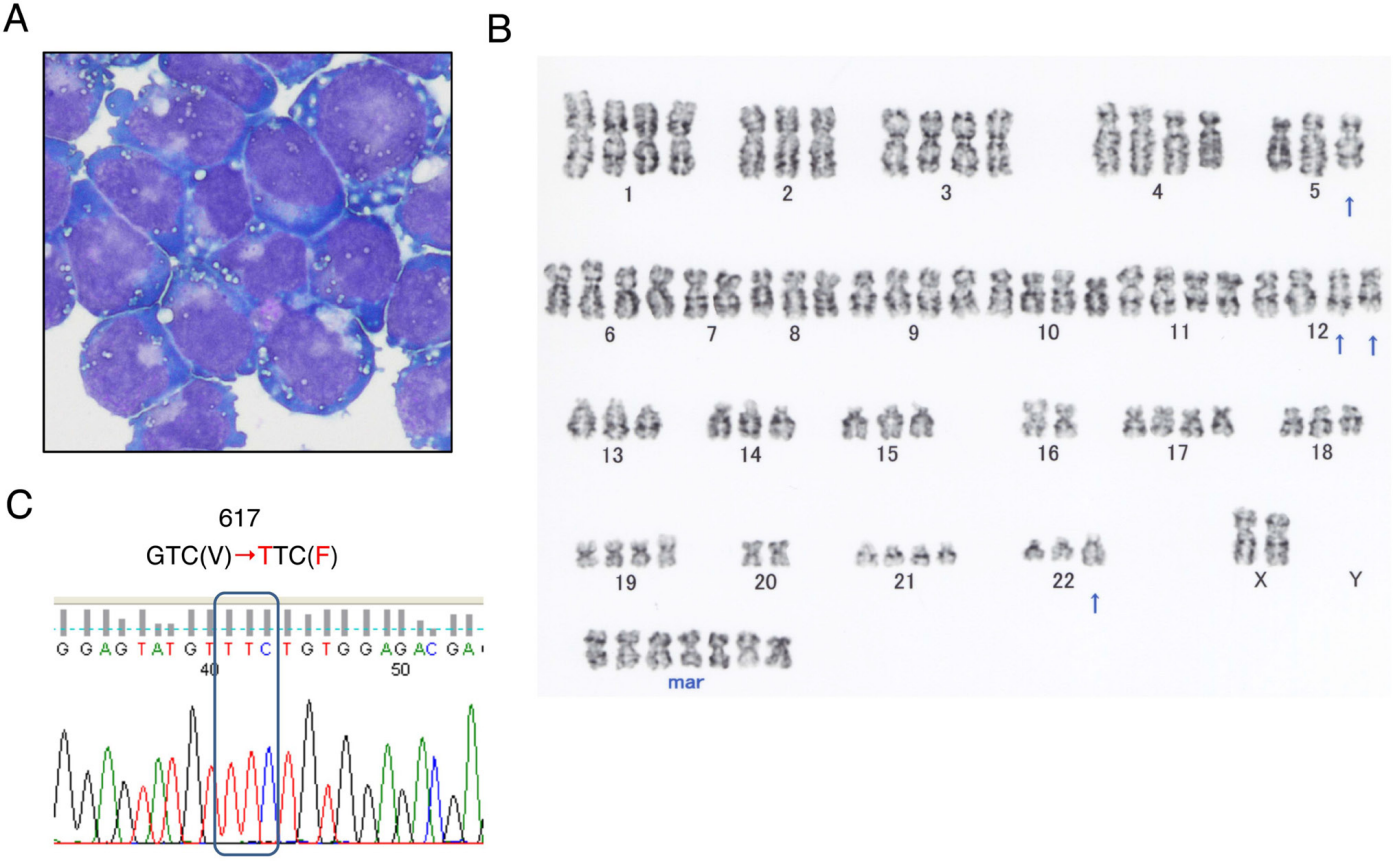
Morphological, cytogenetic, and genetic analyses of PVTL-2 cells (**A**) A cytospin preparation of PVTL-2 cells. May-Grunwald-Giemsa staining. (**B**) Giemsa-banded cytogenetic analysis showing a near-tetraploid consensus karyotype, 83,XX,-X,-X,-2,-5,der(5;7)(p10;q10),-7,-8,del(12)(q?)x2,-13,-14,-15,-16,-16,-18,-20,-20,-22,add(22)(q11.2),+7mar. (**C**) Direct sequence analysis of the JAK2 gene obtained by PCR from PVTL-2 cells. Nucleotide sequences around the codon coding for V617 in normal JAK2 or F617 in the JAK2 mutant are shown with the mutated nucleotide and amino acid sequences indicated in red.

PVTL-2 cells showed round or oval nuclei in fine reticular pattern with several nucleoli and irregular basophilic cytoplasm with vacuoles (Figure 1A), which is similar with the patient's leukemic blasts and PVTL-1 cells established from the same patient [6]. However, the cytoplasm area was narrower and more abundant with vacuoles as compared with primary cells and PVTL-1 cells. PVTL-2 cells were positive for surface CD33, CD71, and CD117, but negative for CD7, CD13, CD19, CD34, CD41, CD56, glycophorin A, HLA-DR, and cytoplasmic MPO. Thus, the immunophenotypes of PVTL-2 cells were partly different from those of PVTL-1, which expressed CD7, CD34, and HLA-DR in addition to CD13 and CD33 at high levels, or primary leukemic cells, which were mostly positive for CD13 or CD34 and partly for CD7 and HLA-DR (Supplementary Table 1) [6]. PVTL-2 cells displayed a near-tetraploid consensus karyotype, 83,XX,-X,-X,-2,-5,der(5;7)(p10;q10),-7,-8,del(12)(q?)x2,-13,-14,-15,-16,-16,-18,-20,-20,-22,add(22)(q11.2),+7mar (Figure 1B), which was quite different from that of PVTL-1 but included some of the common chromosomal abnormalities observed in primary AML cells, such as der(5;7)(p10;q10) and add(22)(q11.2), thus indicating that this cell line was derived from a different subclone of leukemic cells evolved at the transformation to AML. At transformation into AML, the primary leukemic cells were homozygous for the JAK2-V617F mutation [6]. In accordance with this, only the sequences coding for JAK2-V617F were detected in PVTL-2 cells, indicating the cells are homozygous for the JAK2-V617F mutation (Figure 1C), which is also the case with PVTL-1 [6].

### Expression of JAK2-V617F and other signaling and anti-apoptotic molecules in PVTL-1, PVTL-2, and HEL cells

**Figure 2:**
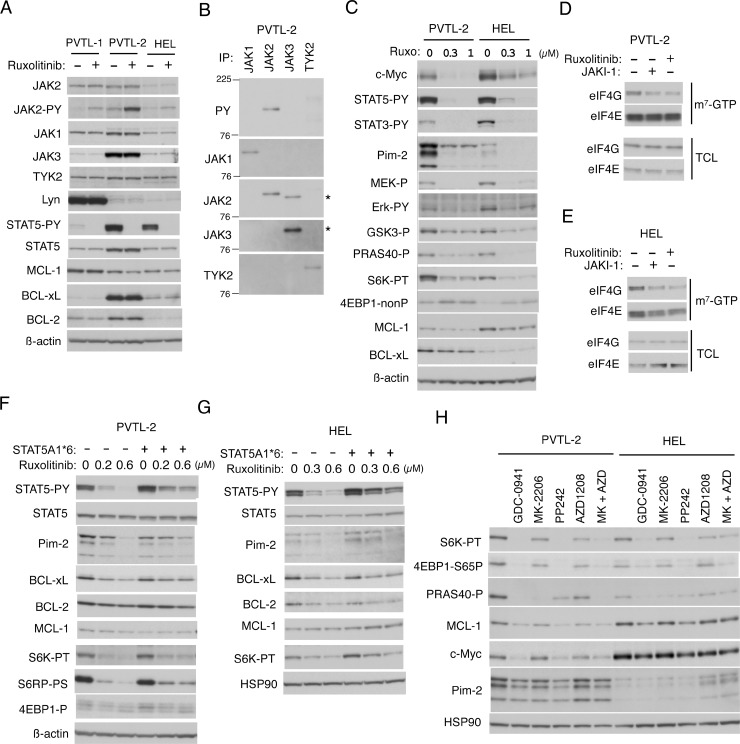
Down stream signaling mechanisms from JAK2-V617F in PVTL-2 and HEL cells (**A**) PVTL-1, PVTL-2, and HEL cells were treated for 3 h with 1 μM ruxolitinib or left untreated as control, as indicated, and subjected to immunoblot analysis using antibodies against indicated proteins. Abbreviations: JAK-PY, phospho-Y1007/1008-JAK2; STAT5-PY, phospho-Y694-STAT5. (**B**) The JAK family members indicated were immunoprecipitated from lysates of PVTL-2 and analyzed by immunoblot analysis using antibodies against indicated proteins. JAK2 was immunoprecipitated with the monoclonal antibody (CS-3230) and was detected using the polyclonal antibody (SC-294), which cross-reacted with JAK3. Positions of JAK3 are indicated by asterisks. PY: anti-phosphotyrosine. (**C**) PVTL-2 or HEL cells were treated with indicated concentrations of ruxolitinib for 6 h and analyzed. Abbreviations: STAT3-PY, phospho-Y705-STAT3; MEK-P, phospho-S217/221-MEK; Erk-PY, phospho-T202/Y204-Erk; GSK3-P, phospho-S9-GSK3ß; PRAS40-P, phospho-T246-PRAS40; S6K-PT, phospho-T389-p70S6 kinase; 4EBP1-nonP, non-phospho-T46-4EBP1. (**D**, **E**) PVTL-2 or HEL cells were treated for 6 h with 1 μM JAKI-1 or 1 μM ruxolitinib, as indicated, and subjected to the cap-binding assay to analyze the eIF4E-eIF4G complex formation. Proteins affinity purified with m^7^-GTP-sepharose (m^7^-GTP) as well as total cell lysates (TCL) were subjected to Western blot analysis. (**F**, **G**) PVTL-2 or HEL cells transduced with STAT5A1^*^6 or vector control cells, as indicated, were treated with indicated concentrations of ruxolitinib for 24 h and subjected to immunoblot analysis. Abbreviations: S6RP-P, phospho-S240/244-S6RP; 4EBP1-P, phospho-T37/46-4EBP1. (**H**) PVTL-2 or HEL cells, as indicated, were treated for 6 h in ASF104 medium with 0.5 μM PP242 or 1 μM of indicated inhibitors and analyzed. Abbreviations: MK, MK-2206; AZD, AZD1208; 4EBP1-S65P, phospho-S65-4EBP1.

In the PVTL-1 cell line we previously established from the same patient, the Src family kinase Lyn was expressed at a very high level and constitutively activated to stimulate the mTORC1 signaling pathway along with JAK2-V617F [6]. Thus, we first examined the expression levels of Lyn and the JAK family kinases in PVTL-2 cells in comparison with PVTL-1 and the JAK2-V617F-positive model cell line HEL, which has been widely used for studies on JAK2-V617F [5]. As we reported previously, Lyn was expressed at a drastically high level in PVTL-1, but at a relatively low level in PVTL-2 as well as in HEL (Figure 2A). On the other hand, JAK3 was expressed at an exceedingly high level in PVTL-2 as compared with PVTL-1 and HEL. JAK2 was expressed at higher levels in PVTL-1 and PVTL-2 than in HEL. The other JAK family kinases JAK1 and TYK2 were expressed at comparable levels in these three cell lines. To confirm that the constitutively activated JAK2 mutant JAK2-V617F is expressed in these cell lines, we treated them with the clinically relevant JAK kinase inhibitor ruxolitinib. As expected, the JAK2 substrate STAT5 was constitutively tyrosine phosphorylated in these cells and was rapidly dephosphorylated by ruxolitinib (Figure 2A). Intriguingly, activation of STAT5 was more robustly observed in PVTL-2 and HEL cells than in PVTL-1 cells, in which Lyn plays a critical role in regulation of proliferation and apoptosis independent of STAT5 [6]. We also confirmed that JAK2 in these cells were constitutively phosphorylated on Y1007/Y1008 in the activation loop and that this phosphorylation was augmented by the type I JAK inhibitor ruxolitinib, which binds the active kinase conformation of JAK2, in accordance with previous reports [6, 24]. To examine if the other JAK family kinases, particularly JAK3 expressed at a remarkably high level, may also be constitutively activated in PVTL-2 cells, we immunoprecipitated these kinases and examined their tyrosine phosphorylation status (Figure 2B). As shown in Figure 2B, although JAK2 was distinctively tyrosine phosphorylated as expected, JAK3 and TYK2 were only very faintly phosphorylated in PVTL-2 cells. Furthermore, activation-specific phospho-tyrosine antibodies against JAK1, JAK3, and TYK2 failed to react with these kinases immunoprecipitated from PVTL-2 (negative data not shown). PVTL-2 expressed BCL-xL and BCL-2 at higher levels than in PVTL-1 and HEL, while MCL-1 was expressed at comparable levels in these cells (Figure 2A). After a short-term treatment with ruxolitinib for 3 h, expression levels of these anti-apoptotic BCL-2 family members did not show any significant changes in these cells (Figure 2A). Thus, unlike PVTL-1, PVTL-2 did not overexpress Lyn but expressed JAK3 and BCL-xL as well as BCL-2 at higher levels as compared with PVTL-1 and HEL.

### STAT5 activated by JAK2-V617F at least partly mediates activation of the mTORC1/4EBP1 pathway and upregulation of BCL-xL, Pim-2, MCL-1, and c-Myc in PVTL-2 and HEL cells

We next examined the downstream signaling events activated by JAK2-V617F in PVTL-2 cells in comparison with HEL cells. As shown in Figure 2C, the MEK/Erk signaling pathway and STAT5 as well as STAT3 were activated by JAK2-V617F in both cell lines, because MEK, Erk, STAT5, and STAT3 were constitutively phosphorylated on activation-specific sites and showed dephosphorylation after treatment with ruxolitinib in these cells. The mTORC1 pathway was also activated by JAK2-V617F in PVTL-2 and HEL cells, because ruxolitinib reduced phosphorylation of p70S6K on T389 and induced dephosphorylation of 4EBP1, well-established substrates of the mTORC1 complex (Figure 2C). We next performed the pull-down assays using m^7^-GTP beads to evaluate the effect of ruxolitinib on formation of the eIF4E/eIF4G complex, which enhances the cap-dependent translation of mRNAs having lengthy, G+C-rich, highly structured 5’-UTRs, such as MCL-1 or c-Myc mRNA [20], and is inhibited by binding of dephosphorylated 4EBP1 with eIF4E. As expected, ruxolitinib as well as the pan-JAK inhibitor JAKI-1 reduced the amount of eIF4G pulled down with eIF4E bound to m^7^-GTP in PVTL-2 and HEL cells (Figure 2D, 2E). In accordance with this, treatment of these cells with ruxolitinib for 6 h reduced expression levels of MCL-1 and c-Myc (Figure 2C).

In addition, ruxolitinib remarkably reduced the expression levels of Pim-2, a serine/threonine kinase known to be transcriptionally upregulated by STAT5 [25], in both PVTL-2 and HEL (Figure 2C). Although the 3 isoforms of Pim-2, created from the alternative translation start sites [26], were expressed at much higher levels in PVTL-2 cells than in HEL cells, the highly unstable two smaller isoforms rapidly disappeared after treatment with a relatively low concentration of ruxolitinib in parallel with dephosphorylation of STAT5 in both cell lines. To confirm that the expression of Pim-2 is regulated through STAT5 by JAK2-V617F in these cells, we expressed a STAT5 mutant, STAT5A1^*^6, which has two mutations, H299R located upstream of the putative DNA binding domain and S711F in the transactivation domain, and is constitutively phosphorylated on tyrosine residues, localized in the nucleus, and transcriptionally active [27], and examined its effects. As shown in Figure 2F and 2G, although the expression level of STAT5 was not distinctively increased in PVTL-2 or HEL cells transduced with STAT5A1^*^6 than in vector control cells, that of tyrosine-phosphorylated form was expressed at a higher level and was less remarkably reduced by ruxolitinib. As expected, expression levels of Pim-2 were also less remarkably affected by ruxolitinib in these cells transduced with STAT5A1^*^6 than in vector control cells. It was also revealed that treatment with ruxolitinib for 24 h decreased the expression level of BCL-2 and, more distinctively, that of BCL-xL in both HEL and PVTL-2 cells and that these effects were at least partly prevented by the expression of STAT5A1^*^6. Moreover, downregulation of MCL-1 expression after treatment with ruxolitinib for 6 h was partly prevented by the expression of STAT5A1^*^6, while that of c-Myc was also partly prevented by the activated STAT5 mutant at least in PVTL-2 cells (Supplementary Figure 1A, 1B). Intriguingly, inhibition of the mTORC1 pathway by ruxolitinib was also partly prevented by STAT5A1^*^6 more distinctively in PVTL-2 cells than in HEL cells expressing Pim-2 at a lower level (Figure 2F, 2G, Supplementary Figure 1A, 1B). Together these results suggest that JAK2-V617F increases expression levels of Pim-2, BCL-xL and probably BCL-2 through activation of STAT5 and may enhance the activation of mTORC1 possibly through Pim kinases expressed by STAT5 to upregulate expression levels of MCL-1 and c-Myc.

Although we could not observe activation-specific phosphorylation of Akt on T308 or S374 (negative data not shown), phosphorylation of GSK3ß on S9 or that of PRAS40 on T246, which is known to be mediated by Akt, was reduced by ruxolitinib in PVTL-2 and HEL cells (Figure 2C) as well as in PVTL-1 cells as we reported previously [6]. Thus, we examined the possible involvement of the PI3K/Akt pathway downstream of JAK2-V617F in activation of the mTORC1 pathway using specific inhibitors for PI3K and Akt. The pan-PI3K inhibitor GDC-0941 strongly inhibited phosphorylation of p70S6K on T389 and 4EBP1 on S65 to reduce expression of MCL-1 to comparable extents with the mTOR inhibitor PP242 or ruxolitinib in both cell lines (Figure 2H and Supplementary Figure 1C, 1D). On the other hand, the Akt inhibitor MK-2206 as well as the pan-Pim kinase inhibitor AZD1208 barely inhibited the mTORC1/MCL-1 pathway when used alone. However, these inhibitors in combination inhibited this pathway to the similar extents with the inhibitor for PI3K or mTOR in PVTL-2. Similar results were obtained with HEL cells, although AZD1208 was less effective in these cells than in PVTL-2 cells, which express Pim-2 at a much higher level. These data support the hypothesis that Pim-2 may cooperatively upregulate the mTORC1 pathway with Akt, which is consistent with the results that STAT5A1^*^6 partially inhibited downregulation of Pim-2 expression and mTORC1 activation in both PVTL-2 and HEL cells treated with ruxolitinib (Figure 2F, 2G, Supplementary Figure 1A, 1B).

Because the phenotypic analysis by flow cytometry revealed that both PVTL-1 and PVTL-2 cells were positive for CD117 (c-Kit), we examined the expression and phosphorylation status of c-Kit in these cells as well as in HEL cells and its possible effect on STAT3 activation, which is known to mediate leukemogenesis downstream of aberrantly activated c-Kit in AML cells [28]. As shown in Supplementary Figure 1E, c-Kit was constitutively tyrosine phosphorylated strongly in PVTL-1 cells, although its expression level in these cells was much lower than in PVTL-2 or HEL cells. c-Kit was expressed at a higher level in PVTL-2 cells and migrated faster than that in other cell lines for some unknown reason but was barely tyrosine phosphorylated. Although the tyrosine kinase inhibitor imatinib, which inhibits c-Kit, moderately reduced the expression and tyrosine phosphorylation levels of c-Kit in these cells, it did not show any effect on tyrosine phosphorylation of STAT3, which was found to be expressed at a much lower level in PVTL-1 cells than in PVTL-2 and HEL cells. Taken together with the data that ruxolitinib abrogated tyrosine phosphorylation of STAT3 in PVTL-2 and HEL cells (Figure 2C), these results indicate that JAK2-V617F but not c-Kit is responsible for tyrosine phosphorylation of STAT3 in these cells.

### Inhibition of JAK2-V617F very effectively reduces proliferation of PVTL-2 and HEL cells without considerably inducing cell death

**Figure 3:**
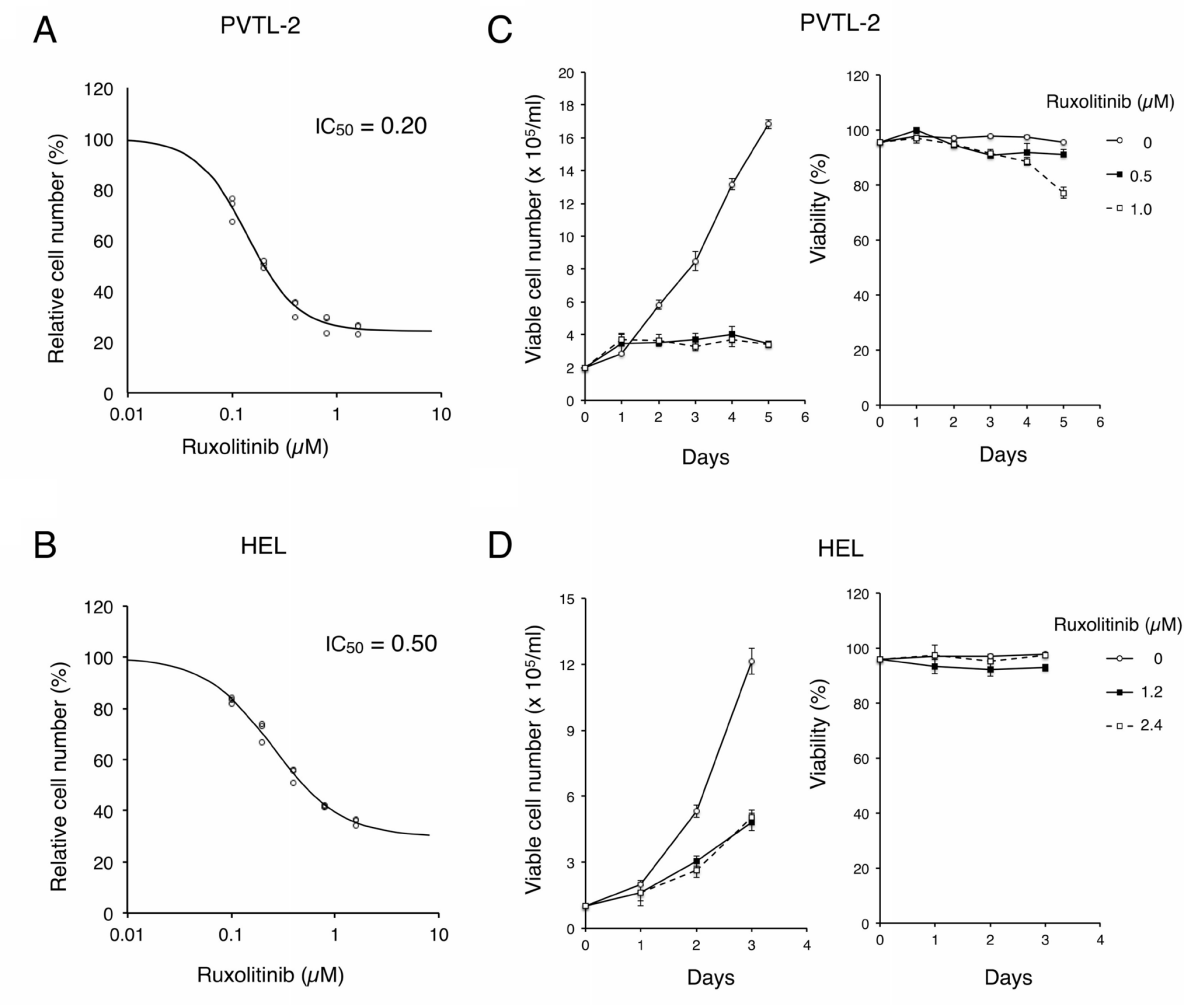
Ruxolitinib effectively inhibits proliferation of PVTL-2 and HEL cells without distinctively inducing cell death (**A**, **B**) PVTL-2 or HEL cells, as indicated, were cultured with indicated concentrations of ruxolitinib for 48 h. Viable cell numbers were measured by the Cell counting Kit-8. Relative cell numbers expressed as percentages of cell numbers without ruxolitinib from triplicate samples are plotted with four-parameter logistic curves obtained by using ImageJ software with calculated IC_50_ (μM) indicated. (**C**, **D**) PVTL-2 or HEL cells, as indicated, were cultured with indicated concentrations of ruxolitinib for indicated days. Viable cell numbers and viability were counted and plotted. Each data point represents the mean of triplicate determinations, with error bars indicating standard errors.

We next evaluated the significance of JAK2-V617F on proliferation and survival of PVTL-2 as well as HEL cells by examining the effects of ruxolitinib and other JAK kinase inhibitors. As shown in Figure 3A and 3B, ruxolitinib very efficiently inhibited proliferation of PVTL-2 cells with a half-inhibitory concentration (IC_50_) of 0.20 μM, while IC_50_ of ruxolitinib for HEL was estimated as 0.50 μM (Figure 3A, 3B). We also examined the sensitivities of PVTL-2 cells to various other JAK family kinase inhibitors in comparison with HEL cells. Whereas these two cell lines showed comparable sensitivities to fedratinib and momelotinib, PVTL-2 was more sensitive than HEL to tofacitinib, which inhibits JAK3 more selectively than other JAK family kinases (Table 1, Supplementary Figure 2). Thus, as compared with HEL cells, PVTL-2 cells showed higher sensitivities to ruxolitinib and tofacitinib and comparable sensitivities to other JAK family kinase inhibitors. As shown in Figure 3C, PVTL-2 cells proliferated with a doubling time of approximately 40 h. It was confirmed that ruxolitinib at 0.5 μM or 1 μM remarkably inhibited proliferation of PVTL-2 cells. However, these cells still survived showing only a modest decline in viability even after treatment with 1 μM ruxolitinib for 5 days. Similarly, ruxolitinib at concentrations up to 4 X IC_50_ inhibited proliferation of HEL cells but barely affected viability up to 3 days (Figure 3D). Thus, inhibition of JAK2-V617F by ruxolitinib very effectively arrested proliferation of PVTL-2 as well as HEL cells without appreciably suppressing survival of these cells.

**Table 1:** IC_50_ (μM) of JAK kinase inhibitors for PVTL-2 and HEL cells

	PVTL-2	HEL
Ruxolitinib	0.20	0.50
Fedratinib	1.44	1.39
Momelotinib	2.40	1.93
Tofacitinib	2.08	8.86

### Inhibition of JAK2-V617F and BCL-xL/BCL-2 synergistically induce caspase-dependent apoptosis involving activation of Bak and Bax in HEL cells but not in PVTL-2 cells

**Figure 4:**
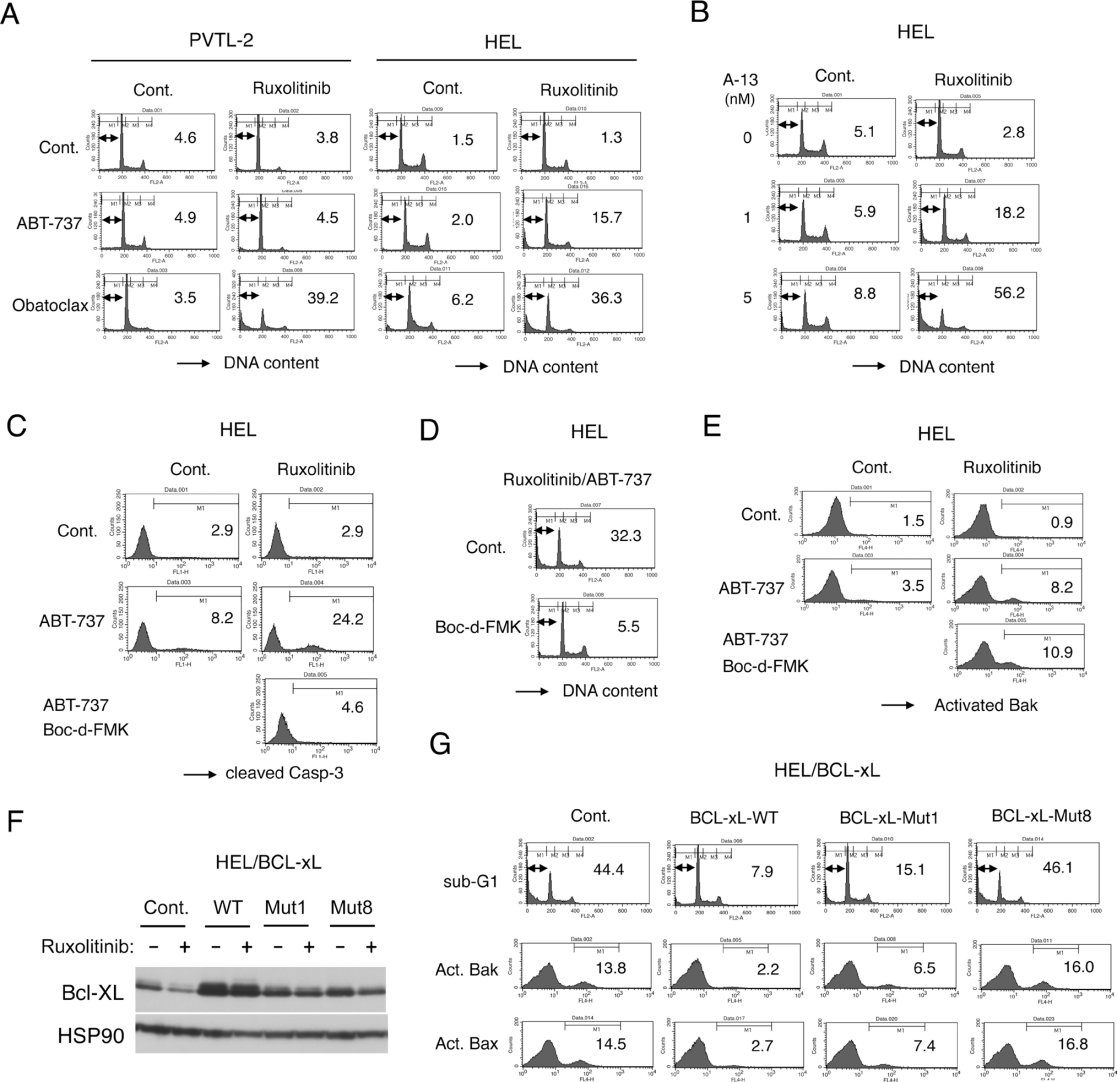
Ruxolitinib induces apoptosis synergistically with obatoclax in PVTL-2 cells and with ABT-737 or obatoclax in HEL cells through caspase-dependent mechanisms involving activation of Bax and Bak (**A**) PVTL-2 or HEL cells, as indicated, were cultured with 1 μM ABT-737 or 0.5 μM obatoclax in the presence or absence of 1 μM ruxolitinib, as indicated, for 48 h and analyzed for the cellular DNA content by flow cytometry. Percentages of apoptotic cells with sub-G1 DNA content are indicated. (**B**) HEL cells were cultured with indicated concentrations of A-1331852 (A-13) in the presence or absence of 1 μM ruxolitinib, as indicated, for 48 h and analyzed. (**C**) HEL cells were cultured with 1 μM ABT-737 and 100 μM Boc-d-FMK in the presence or absence of 1 μM ruxolitinib, as indicated, for 24 h and analyzed for activation of Caspase-3 by flow cytometry. Percentages of cells with cleaved Caspase-3 are indicated. (**D**) HEL cells were cultured with 1 μM ruxolitinib and 1 μM ABT-737 in the presence or absence of 100 μM Boc-d-FMK, as indicated, for 48 h and analyzed for the cellular DNA content. (**E**) HEL cells were cultured with 1 μM ABT-737, 100 μM Boc-d-FMK, and 1 μM ruxolitinib, as indicated, for 16 h and analyzed for activation of Bak by flow cytometry. Percentages of cells with activated Bak are indicated. (**F**) HEL cells transduced with the wild-type BCL-xL (WT) or its mutants (Mut1, Mut8) and vector control cells (Cont.) were cultured for 24 h with or without 1 μM ruxolitinib, as indicated, and subjected to immunoblot analysis using antibodies against indicated proteins. (**G**) HEL cells transduced with the wild-type BCL-xL or its mutants (Mut1, Mut8) and vector control cells, as indicated, were treated with the combination of 1 μM ruxolitinib and 1 μM ABT-737 for 48 h or 16 h to be subjected to flow cytometric analyses for the cellular DNA content or activation of Bax and Bak, respectively. Percentages of apoptotic cells with sub-G1 DNA content and activated Bak (Act. Bak) or Bax (Act. Bax) are indicated.

We confirmed that inhibition of JAK2-V617F by ruxolitinib alone did not significantly induce apoptosis in PVTL-2 or HEL cells, as examined by flow cytometry for appearance of cells with sub-G1 DNA content, a hallmark of apoptotic cells (Figure 4A). We next examined whether inhibition of the anti-apoptotic BCL-2 family molecules may overcome resistance of these cells to ruxolitinib for induction of apoptosis. We first examined effects of the BH3 mimetic ABT-737 [18], mainly inhibiting BCL-xL and BCL-2, and obatoclax, putatively inhibiting MCL-1 as well as BCL-xL and BCL-2 [29]. As shown in Figure 4A, ABT-737 alone at 1 μM also did not significantly induce apoptosis in PVTL-2 or HEL cells. However, when combined with ruxolitinib, ABT-737 induced apoptosis distinctively in HEL cells but not in PVTL-2 cells. On the other hand, obatoclax induced apoptosis synergistically with ruxolitinib in both HEL and PVTL-2 cells. We also examined effects of the BH3 mimetic navitoclax or venetoclax, which is under development for clinical usages or clinically approved for treatment of chronic lymphocytic lymphoma, respectively [18]. As expected, apoptosis was induced synergistically with ruxolitinib in HEL cells by navitoclax, which inhibits both BCL-xL and BCL-2 in a similar manner with ABT-737, but not by the BCL-2-specific inhibitor venetoclax (Supplementary Figure 3A). On the other hand, both BH3 mimetics failed to induce apoptosis synergistically with ruxolitinib in PVTL-2 cells. Finally, the BH3 mimetic specific for BCL-xL A-1331852 [18] was demonstrated to induce apoptosis synergistically with ruxolitinib in HEL cells (Figure 4B). It was further confirmed that A-1331852 and ruxolitinib synergistically reduced viable cell numbers of HEL cells, as judged by combination index (CI) values obtained by the method of Chuo and Talalay [30] being less than 1 at all the concentrations examined (Supplementary Figure 3B). These results indicate that inhibition of mainly BCL-xL is sufficient to overcome resistance of HEL cells to ruxolitinib for induction of apoptosis and suggest the possibility that inhibition of MCL-1 in addition to BCL-xL/BCL-2 might have been required to overcome the resistance of PVTL-2 cells.

We next examined involvement of the pro-apoptotic BCL-2 family effector molecules Bak and Bax as well as caspase activation in induction of apoptosis in these cells. As shown in Figure 4C, whereas the cleavage or activation of Caspase-3 was induced only modestly by ruxolitinib or ABT-737 in HEL cells, it was prominently induced by the combined treatment, which correlated with induction of apoptosis in these cells. Similarly, ruxolitinib in combination with obatoclax synergistically induced activation of Caspase-3 prominently in PVTL-2 cells in parallel with induction of apoptosis, whereas ruxolitinib or obatoclax alone only modestly activated Caspase-3 (Supplementary Figure 3C). Furthermore, the pan-caspase inhibitor Boc-d-FMK mostly prevented both activation of Caspase-3 and induction of apoptosis induced by the combined treatment in HEL and PVTL-2 cells (Figure 4C, 4D, Supplementary Figure 3C, 3D), thus indicating that the induction of apoptosis is dependent on activation of caspases. Using an anti-Bax antibody specifically reactive with its activated form, we found that Bax was activated only after the combined treatment with ABT-737 and ruxolitinib in HEL cells or obatoclax and ruxolitinib in PVTL-2 cells but not after treatment with any of these inhibitors alone (Supplementary Figure 3E, 3F). Moreover, not only Bax but also Bak was activated by the combined treatment of HEL cells with ruxolitinib and ABT-737 and that activation of Bak as well as Bax was not inhibited by Boc-d-FMK, thus indicating that activation of these pro-apoptotic BCL-2 family members was not caused subsequently by activated caspases in cells undergoing apoptosis (Figure 4E, Supplementary Figure 3E, 3F). To confirm that the synergistic effect of ABT-737 with ruxolitinib in HEL cells is mainly mediated through inhibition of BCL-2/BCL-xL but not through off target effects and to explore the molecular mechanisms involved more precisely, we overexpressed wild-type BCL-xL, the BCL-xL-Mut1 mutant with F131V and D133A substitutions defective specifically in binding with Bax and Bak, or the BCL-xL-Mut8 mutant with G183E, R139L, and I140N substitutions defective also in binding with the BH3-only pro-apoptotic proteins [31, 32]. We first confirmed that the expression level of wild-type BCL-xL as well as its mutants were higher than that of endogenous BCL-xL in vector control cells and was less remarkably reduced after 24 h treatment with ruxolitinib unlike that of endogenous BCL-xL (Figure 4F). As shown in Figure 4G, overexpression of BCL-xL or, to a slightly lesser extent, BCL-xL-Mut1 inhibited the synergistic induction of apoptosis in HEL cells induced by the combined treatment with ABT-737 and ruxolitinib. On the other hand, BCL-xL-Mut8, expressed at a comparable level with BCL-xL-Mut1, did not show any inhibitory effects to suppress apoptosis induced by ABT-737 and ruxolitinib. These results imply that the synergistic effect of ABT-737 with ruxolitinib in HEL cells should be indeed mediated mainly through inhibition of BCL-xL to bind with the BH3-only pro-apoptotic BCL-2 family members, such as Bim, but not with the pro-apoptotic effector proteins Bak and Bax.

### Inhibition of autophagy mainly mediates the effect of obatoclax to induce apoptosis synergistically with inhibition of JAK2-V617F in PVTL-2 cells

**Figure 5:**
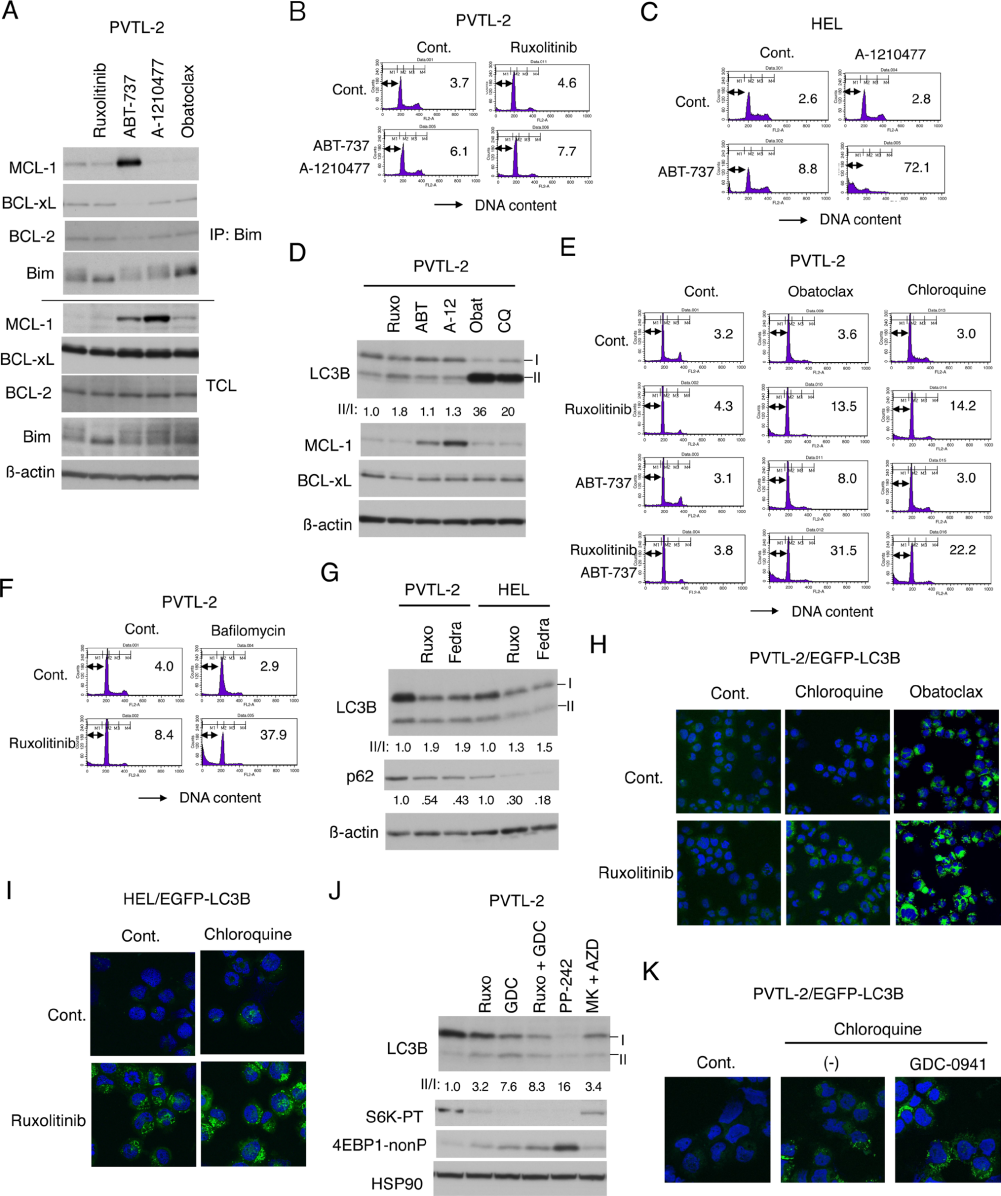
Inhibition of autophagy induced by ruxolitinib leads to induction of apoptosis in PVTL-2 cells (**A**) PVTL-2 cells were cultured with 1 μM ruxolitinib, 1 μM ABT-737, 10 μM A-1210477, or 0.5 μM obatoclax, as indicated, for 8 h and lysed. Immunoprecipitates (IP) obtained with anti-Bim and total cell lysates (TCL) were subjected to immunoblot analysis using antibodies against indicated proteins. (**B**) PVTL-2 cells were cultured with 1 μM ABT-737, 10 μM A-1210477, and 1 μM ruxolitinib, as indicated, for 48 h and analyzed for the cellular DNA content by flow cytometry. Percentages of apoptotic cells with sub-G1 DNA content are indicated. (**C**) HEL cells were cultured with 1 μM ABT-737 and 10 μM A-1210477, as indicated, for 48 h and analyzed. (**D**) PVTL-2 cells were treated with 1 μM ruxolitinib (Ruxo), 1 μM ABT-737 (ABT), 10 μM A-1210477 (A-12), 0.5 μM obatoclax (Obat), or 40 μM chloroquine (CQ) for 8 h and subjected to immunoblot analysis using antibodies against indicated proteins. Positions of LC3B-I and LC3B-II as well as the relative ratio of LC3B-II/LC3B-I (II/I) determined by densitometric analysis are indicated. (**E**) PVTL-2 cells were cultured with 1 μM ruxolitinib, 1 μM ABT-737, 0.5 μM obatoclax, or 40 μM chloroquine, as indicated, for 36 h and analyzed for the cellular DNA content by flow cytometry. Percentages of apoptotic cells with sub-G1 DNA content are indicated. (**F**) PVTL-2 cells were cultured with 1 μM ruxolitinib and 100 nM bafilomycin A1, as indicated, for 48 h and analyzed. (**G**) PVTL-2 or HEL cells were treated with 1.5 μM ruxolitinib (Ruxo) or 1.5 μM fedratinib (Fedra), as indicated, for 16 h and subjected to immunoblot analysis using antibodies against indicated proteins. Relative expression levels of p62 were determined by densitometric analysis and are shown below the panel. (**H**) PVTL-2 cells transduced with EGFP-LC3B were treated with or without 1 μM ruxolitinib for 6 h with or without 40 μM chloroquine or 0.5 μM obatoclax added during the last 2 h. Cells were stained with DAPI for nuclear staining and analyzed by confocal immunofluorescence microscopy. Representative merged images of EGFP-LC3B (green) and DAPI (blue) are shown. (**I**) HEL cells transduced with EGFP-LC3B were treated with or without 1 μM ruxolitinib for 4 h in the presence or absence of 50 μM chloroquine and analyzed by confocal immunofluorescence microscopy. (**J**) PVTL-2 cells were treated for 6 h with 1 μM of ruxolitinib (Ruxo), GDC-0941 (GDC), PP242, MK-2206 (MK), or AZD1208 (AZD), as indicated, and analyzed. Abbreviations: S6K-PT, phospho-T389-p70S6 kinase; 4EBP1-nonP, non-phospho-T46-4EBP1. (**K**) PVTL-2 cells transduced with EGFP-LC3B were left untreated for control or treated with or without 1 μM GDC-0941 for 6 h with 40 μM chloroquine added during the last 2 h and analyzed by confocal immunofluorescence microscopy.

To explore the possibility that MCL-1 may play a critical role in prevention of apoptosis in ruxolitinib-treated PVTL-2 cells, which undergo apoptosis synergistically with the putative pan-BH3 mimetic obatoclax but not with ABT-737, we next examined effects of the MCL-1 specific BH3 mimetic A-1210477 [33]. ABT-737 inhibited the binding of BCL-xL as well as BCL-2 with the BH3-only pro-apoptotic protein Bim in PVTL-2 cells, as expected, and increased the expression level of MCL-1 without affecting its ability to bind Bim (Figure 5A). The increased level of MCL-1 expression could be explained by its stabilization through binding of Bim released from BCL-xL and BCL-2 to the BH3-binding groove of MCL-1, the region involved in its degradation through the ubiquitin/proteasomal pathway. Although the expression level of MCL-1 was also increased drastically by A-1210477, most likely by blocking the BH3-binding groove of MCL-1, the amount of MCL-1 bound to Bim was not increased, indicating that A-1210477 indeed inhibited MCL-1 to bind the BH3 domain-containing proteins. On the other hand, A-1210477 did not show any significant effect on BCL-xL or BCL-2. These effects of ABT-737 and A-1210477 were similarly observed in HEL cells (Supplementary Figure 4A). As compared with A-1210477, obatoclax showed much less significant effects on the expression level of MCL-1 and its binding with Bim in PVTL-2 cells and did not apparently inhibit the binding of Bim with BCL-xL or BCL-2 at the concentration demonstrated to induce apoptosis synergistically with ruxolitinib in these cells (Figure 5A). These results indicate that obatoclax inhibited MCL-1 much less significantly than A-1210477 and that neither obatoclax nor A-1210477 significantly affected BCL-xL and BCL-2 in these cells.

We next examined whether combined treatment with ABT-737 and A-1210477 may induce apoptosis synergistically with ruxolitinib in PVTL-2 cells in a similar manner with treatment with obatoclax. However, inhibition of MCL-1 and BCL-xL/BCL-2 by the combined treatment failed to induce apoptosis synergistically with inhibition of JAK2-V617F in these cells (Figure 5B). On the other hand, A-1210477 drastically induced apoptosis synergistically with ABT-737 in HEL cells, while it induced apoptosis less significantly than ABT-737 in combination with ruxolitinib (Figure 5C, Supplementary Figure 4B). As expected, overexpression of wild-type BCL-xL and, to a lesser extent, BCL-xL-Mut1, but not BCL-xL-Mut8, reduced apoptosis induced synergistically with ABT-737 and A-1210477 (Supplementary Figure 4C). Together, these results indicate that inhibition of BCL-xL/BCL-2 and MCL-1 along with that of JAK2-V617F was not sufficient to induce apoptosis in PVTL-2 cells, whereas inhibition of all these anti-apoptotic BCL-2 family members was sufficient to induce apoptosis significantly in HEL cells.

Therefore, we explored the effects of obatoclax in PVTL-2 cells further and found that it drastically increased the expression level of LC3B-II (Figure 5D) in a similar manner with the autophagy inhibitor chloroquine, which is in accordance with previous studies reporting that obatoclax inhibited the late stage of autophagy [34, 35]. Moreover, chloroquine induced apoptosis synergistically with ruxolitinib in PVTL-2 cells in a similar manner with obatoclax and overcame the resistance of these cells to combined treatment with ABT-737 and ruxolitinib, albeit to a lesser degree than obatoclax did (Figure 5E). Consistent with this, apoptosis was also induced synergistically in PVTL-2 cells by treatment with ruxolitinib and bafilomycin A1, which inhibits the late stage of autophagy similarly with chloroquine (Figure 5F). Although chloroquine failed to notably induce apoptosis with ruxolitinib in HEL cells, it remarkably enhanced apoptosis induced by combined treatment with ABT-737 and ruxolitinib in these cells, thus suggesting that autophagy may be negatively regulating apoptosis also in HEL cells when JAK2-V617F and BCL-2/BCL-xL were inhibited (Supplementary Figure 4D).

To explore the significance of autophagy on induction of apoptosis, we next examined whether autophagy may be induced by inhibition of JAK2-V617F in these cells. Ruxolitinib increased the LC3B-II/I ratio with or without co-treatment later with chloroquine and decreased the expression level of p62 in both PVTL-2 and HEL cells, which indicates that ruxolitinib enhanced the autophagic flow in these cells (Figure 5D, 5G, Supplementary Figure 4E, 4F). Inhibition of JAK2-V617F by fedratinib also showed similar effects with that by ruxolitinib in both PVTL-2 and HEL cells (Figure 5G). Autophagy induced by ruxolitinib was at least not downregulated by co-treatment with ABT-737 (Supplementary Figure 4F, 4G). Consistent with these data, it was demonstrated by confocal microscopy that ruxolitinib increased the GFP-LC3 punctuation/aggregation, most significantly when cells were co-treated later with chloroquine, in both PVTL-2 and HEL cells expressing the fusion protein and that obatoclax strongly induced the GFP-LC3 punctuation/aggregation in PVTL-2 cells, which was significantly enhanced by co-treatment with ruxolitinib (Figure 5H, 5I). To explore the mechanisms involved in induction of autophagy by inhibition of JAK2-V617F, we next examined the possible effects of inhibitors of the PI3K/Akt/mTOR pathway. Inhibition of this pathway with GDC-0941, PP242, or combination of MK-2206 and AZD1208 increased the LC3B-II/I ratio in both PVTL-2 and HEL cells in similar manners with ruxolitinib, which correlated with inhibition of the mTORC1 activity (Figure 5J, Supplementary Figure 4G). As shown in Figure 5K, it was confirmed that GDC-0941 enhanced the GFP-LC3 punctuation/aggregation in PVTL-2 cells treated later with chloroquine. Furthermore, the Akt inhibitor MK-2206 enhanced the autophagic flux in HEL cells in 4 h without affecting proliferation of these cells in 48 h (Supplementary Figure 4H, 4I). MK-2206 enhanced autophagy also in PVTL-2 cells in 6 h, while only modestly reducing proliferation in 48 h (Supplementary Figure 4J, 4K). On the other hand, The MEK inhibitor PD98059 at 100 μM slightly reduced proliferation but did not at all enhance autophagy in PVTL-2 cells. Thus, it is not plausible that the enhanced autophagy is simply a general response of these cells to inhibition of proliferation. Together, these results suggest that inhibition of the mTORC1 pathway downstream of JAK2-V617F by ruxolitinib may induce autophagy in PVTL-2 as well as HEL cells and that obatoclax may induce apoptosis synergistically with ruxolitinib mainly through inhibition of autophagy induced by ruxolitinib and possibly in part through inhibition of MCL-1.

## DISCUSSION

In the first part of present study, we have reported establishment of the new JAK2-V617F-positive cell line PVTL-2 from the same patient from whom we previously established PVTL-1 [6]. However, these cell lines are immunophenotypically and karyotypically different from each other and supposed to be derived from different subclones of leukemic cells evolving at the transformation of PV into myelodysplastic syndrome (MDS)/AML based on cytogenetic data. Nevertheless, both PVTL-1 and PVTL-2 cells are homozygous for the JAK2-V617F mutation and exhibited losses of long-arm material from both chromosomes 5 and 7 in common with previously reported 5 JAK2-V617F-positive cell lines, except for SET-2 cells heterozygous for JAK2-V617F and UKE-1 cells showing monosomy 7 alone [5]. Thus, these common features might have contributed to transformation of MPNs into MDS/AML or to establishment as a cell line, which needs to be explored in future studies. On the other hand, PVTL-1 cells are exceptional in over expressing Lyn and dependent on this Src family kinase in addition to JAK2-V617F [6]. In this regard, PVTL-2 cells expressed JAK3 at a much higher level than PVTL-1 or HEL cells (Figure 2A). However, JAK3 did not show the activation specific phosphorylation on Y980/981 in PVTL-2 cells (negative data not shown). Moreover, although PVTL-2 showed a higher sensitivity than HEL to tofacitinib, which preferentially inhibits JAK3, PVTL-2 was also more sensitive than HEL to the JAK1/JAK2 inhibitor ruxolitinib (Table 1). Thus, it is unlikely that JAK3 is constitutively activated in PVTL-2 cells, which should thus depend solely on JAK2-V617F.

Previous studies, including ours, have shown that the mTORC1 pathway is activated in JAK2-V617F-positive cells to play a role mainly in promotion of cell proliferation [6, 15]. However, the mechanisms for activation have remained elusive partly because of the difficulty in detecting activation-specific phosphorylation of Akt by immunoblotting in most of the JAK2-V617F-positive post-MPN sAML cell lines, including PVTL-1, PVTL-2, and HEL cells [6, 36]. In the present study, we have revealed that STAT5 activated by JAK2-V617F not only upregulated expression of Pim-2 and BCL-xL, products of target genes of STAT5 [25, 37], but also enhanced the mTORC1/p70S6K/eIF4F pathway possibly leading to upregulation of MCL-1 as well as c-Myc. This is because the activated STAT5 mutant partly prevented ruxolitinib-induced inhibition of mTORC1 as well as downregulation of MCL-1 and c-Myc, particularly in PVTL-2 cells expressing Pim-2 at a higher level than HEL cells (Figure 2F, 2G, Supplementary Figure 1A, 1B). This is in accordance with a previous report on a JAK2-V617F murine model, in which deficiency of STAT5 failed to enhance phosphorylation of p70S6 kinase [38], although the mechanisms involved were not examined. In the present study, we revealed that the pan-Pim kinase inhibitor AZD1208 inhibited the mTORC1 pathway cooperatively with the Akt inhibitor MK-2206 to downregulate expression of MCL-1 and c-Myc, which was also observed more distinctively in PVTL-2 cells than HEL cells (Figure 2H). We have also very recently revealed that Pim kinases are involved downstream of STAT5 in enhancing mTORC1/MCL-1 pathway in FLT3-ITD-expressing AML cells to confer these cells resistance to PI3K/Akt inhibitors [39]. Together, these data suggest that JAK2-V617F activates the mTORC1 pathway cooperatively through the PI3K/Akt and STAT5/Pim kinase pathways. In this regard, Pim kinases have been reported to upregulate the mTORC1 pathway through various mechanisms involving phosphorylation of TSC2 on S1798 [40], PRAS40 on T246 [41], and 4EBP1 on S65 [42] as well as inhibition of AMPK [43]. Future studies are warranted to elucidate the exact molecular mechanisms of activation of mTORC1 through Pim kinases and the significance on cell survival and proliferation to evaluate Pim kinases as well as mTORC1 as molecular targets for new therapies against MPNs and their transformed diseases.

The present study has shown that both PVTL-2 and HEL cells are dependent on JAK2-V617F as expected, because its inhibition by ruxolitinib very efficiently suppressed proliferation of these cells (Figure 3A, 3B). However, viability of these cells was barely affected by treatment with ruxolitinib at concentrations up to five times higher than IC_50_ for cell proliferation (Figure 3C, 3D). Thus, these cells are only marginally dependent on JAK2-V617F for survival, which may contribute to the constant development of cells persistently proliferating in the presence of JAK inhibitors after a long-term culture of various JAK2-V617F-positive AML cell lines in gradually increasing concentrations of ruxolitinib or other JAK inhibitors [9–12]. This may also explain the very limited effect of ruxolitinib for post-MPN sAML [7], from which these cell lines have been derived, as well as the inefficiency of ruxolitinib to reduce the allelic burden of JAK2-V617F mutation and to eradicate neoplastic cells with this mutation in treatment of MPNs [4]. Future studies using primary leukemic cells from post-MPN sAML and hematopoietic progenitor cells from patients with MPNs are warranted to address this possibility and to develop efficient novel therapies for these intractable diseases.

**Figure 6:**
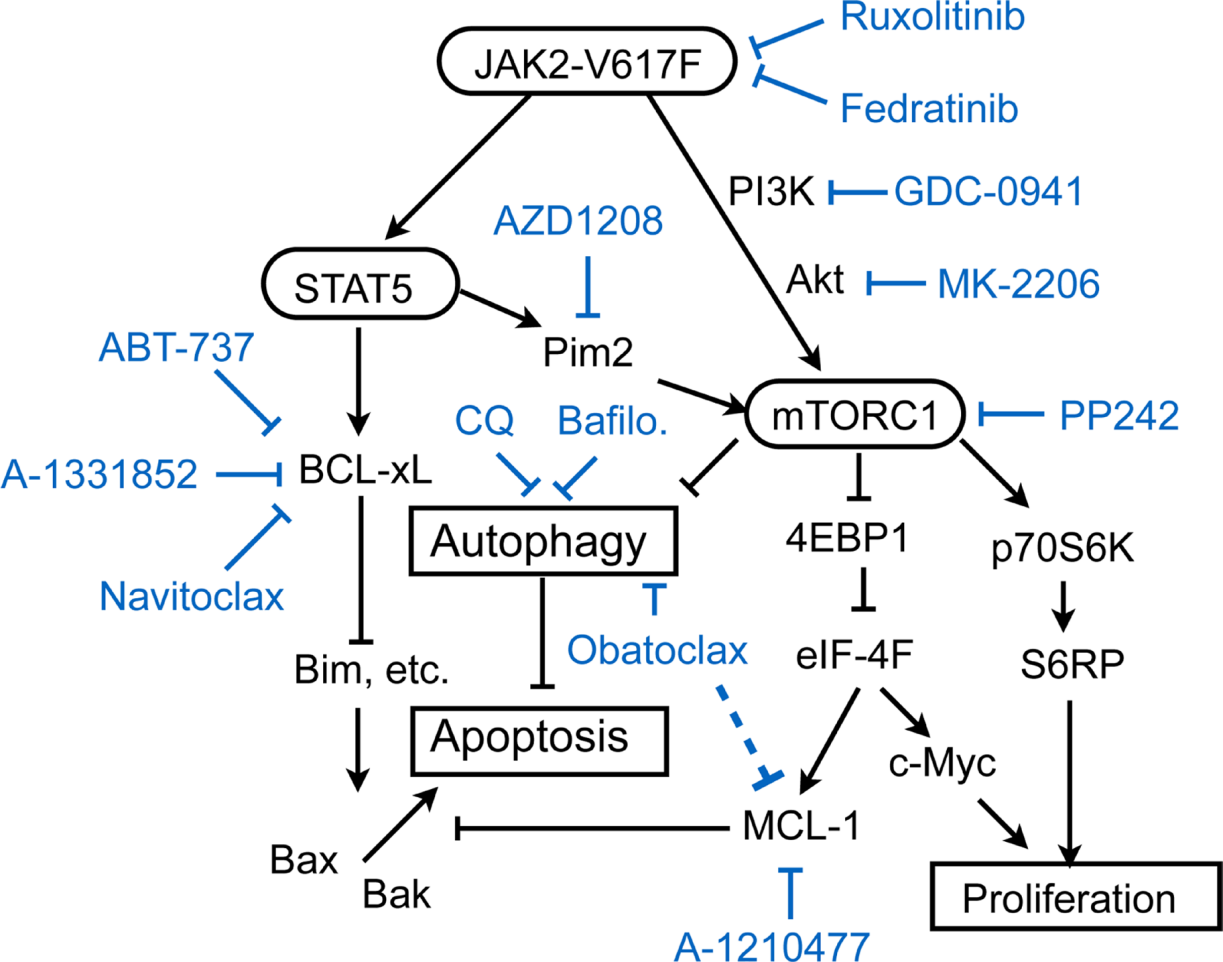
A schematic model of intracellular signaling mechanisms by which JAK2-V617F regulates proliferation, autophagy, and apoptosis in leukemic cells and their inhibition by various small molecule inhibitors Abbreviations: CQ, chloroquine; Bafilo., bafilomycin A1.

In accordance with previous studies in HEL and SET-2 cells [12, 44], we have shown that inhibition of BCL-2/BCL-xL by ABT-737 or navitoclax overcame the resistance of HEL cells to induce caspase-dependent apoptosis involving activation of Bak and Bax in synergistic manners with ruxolitinib (Figure 4C–4EC). Furthermore, inhibition of BCL-xL by its specific BH3 mimetic A-1331852 was enough to induce apoptosis synergistically with ruxolitinib (Figure 4B), while overexpression of BCL-xL prevented apoptosis induced by combined treatment with the BCL-xL/BCL-2 inhibitor ABT-737 and ruxolitinib (Figure 4G). Thus, BCL-xL remaining at a low level after treatment with ruxolitinib may play an essential role in survival of HEL cells. Moreover, BCL-xL-Mut1 defective specifically in binding with Bak/Bax but not BCL-xL-Mut8 defective also in binding with the pro-apoptotic BH3-only proteins [31, 32] protected HEL cells treated in combination with ruxolitinib and ABT-737 from induction of apoptosis involving activation of Bak and Bax (Figure 4G). This suggests that BCL-xL may prevent apoptosis in ruxolitinib-treated HEL cells mainly by sequestering the pro-apoptotic BH3-only proteins, such as Bim, to prevent activation of Bak and Bax (Figure 6). Intriguingly, ABT-737 synergistically induced apoptosis not only with ruxolitinib but also with A-1210477 in HEL cells. Thus, HEL cells should be highly dependent on the anti-apoptotic BCL-2 proteins, and JAK2-V617F may play roles in regulation of expression levels of these and BH3-only proteins and of their interaction in these cells (Figure 6). In this regard, ruxolitinib increased the expression level of Bim in HEL cells or its faster-migrating non-phosphorylated form in PVTL-2 cells in accordance with previous reports [36, 45], which may contribute to enhanced binding of Bim to inhibit BCL-xL as well as MCL-1 (Figure 5A, Supplementary Figure 4A). Moreover, Pim kinases upregulated by JAK2-V617F in SET-2 cells have been described to phosphorylate the BH3-only protein Bad, which is implicated in inhibition of its pro-apoptotic activity [46]. Further studies are required to evaluate expression levels and modification of other BH3-only proteins and their interaction with the anti-apoptotic BCL-2 proteins in cells treated with ruxolitinib.

On the other hand, inhibition of BCL-xL/BCL-2 and MCL-1 by treatment with ABT-737 and A-1210477 failed to induce apoptosis in PVTL-2 alone or in combination with ruxolitinib (Figures 4A, 5B), which may be correlated with the high expression level of BCL-xL in PVTL-2 cells (Figure 2A). Nevertheless, the putative pan-BH3 mimetic obatoclax induced apoptosis synergistically with ruxolitinib in PVTL-2 cells. Further experiments revealed that obatoclax inhibited the late stage of autophagy in PVTL-2 cells (Figure 5D, 5H), in accordance with recent studies in various cell types [34, 35], and that ruxolitinib induced autophagy most likely through inhibition of mTORC1 in PVTL-2 as well as HEL cells (Figure 5G–5KG, Supplementary Figure 4E–4G). Moreover, inhibition of the late stage of autophagy by chloroquine or bafilomycin A1 induced apoptosis synergistically with ruxolitinib in PVTL-2 cells and overcame the resistance to the combined treatment with ruxolitinib and BH3 mimetics to induce apoptosis prominently (Figure 5E, 5F). Inhibition of autophagy also enhanced apoptosis induced by the combined treatment with ruxolitinib and BH3 mimetics in HEL cells (Supplementary Figure 4D). Ruxolitinib has previously been reported to induce or decrease autophagy in chronic myeloid leukemia cells or bladder cancer cells, respectively [47, 48], which implies that it may have different effects on autophagy dependent on cellular context. Although its effects on autophagy in JAK2-V617F-positive neoplastic cells has not been reported to the best of our knowledge, withdrawal of IL-3, which should inactivated JAK2, was shown to induce autophagy in IL-3-dependent hematopoietic cells [49]. Moreover, inhibition of autophagy by chloroquine or by genetic methods led to cell death in IL-3-deprived cells, which is concordant with our results in JAK2-V617F-positive cells. Together, our results suggest that autophagy induced after inhibition of JAK2-V617F as well as anti-apoptotic BCL-2 family proteins remaining at low levels may protect JAK2-V617F-positive cells from induction of apoptosis with divergent efficiencies (Figure 6). Thus, BCL-xL expressed at a low level may play a critical role in prevention of apoptosis in HEL cells treated with ruxolitinib, while induction of autophagy may efficiently protect PVTL-2 cells after inhibition of JAK2-V617F. The differences observed between HEL and PVTL-2 may reflect the diversity of post-MPN sAML, and further studies in various cells, including primary leukemic cells, should shed more light on development of new therapeutic strategies against this disease with dire prognosis.

## MATERIALS AND METHODS

### Establishment of the PVTL-2 cell line and its characterization

Peripheral blood was taken at the same time from the same patient from whom PVTL-1 was derived at the diagnosis of leukemic transformation in April 2010 [6]. Mononuclear cells were separated and cultured as described previously [6]. From a separate culture form PVTL-1, cells started to grow and were confirmed to grow again after conventional freeze-thaw procedure. Morphological, immunophenotypic, and cytogenetic analyses of PVTL-2 cells as well as analysis of the JAK2-V617F mutation in PVTL-2 cells by the allele-specific PCR and direct sequencing methods were performed as described previously [6]. The cells were confirmed to be different from PVTL-1 both immunophenotypically and cytogenetically and, thus, considered to be established as an independent cell line from PVTL-1 to be designated as PVTL-2 in May 2016.

The study was approved by the ethical committee of Tokyo Medical and Dental University. Written informed consent was obtained from the patient in compliance with the Declaration of Helsinki.

### Cells and reagents

PVTL-1 cells and HEL cells were described previously and cultured in RPMI1640 medium with 10% FCS as described [6]. For some experiments, cells were cultured in ASF104 medium (Ajinomoto, Tokyo, Japan) without FCS as indicated.

The pan-JAK inhibitor JAK inhibitor 1 (JAKI-1), the pan-BH3 mimetic obatoclax, and the BCL-2-specific mimetic venetoclax (ABT-199) were purchased from LC laboratories (Woburn, MA). The JAK1/2 inhibitor ruxolitinib and the MEK inhibitor PD98059 were purchased from Calbiochem (La Jolla, CA, USA). The pan-class I PI3K inhibitor GDC-0941 (Pictilisib) and the pan-Pim inhibitor AZD-1208 were purchased from Active Biochem (Kowloon, Hong Kong). The tyrosine kinase inhibitor imatinib was kindly provided by Novartis (Basel, Switzerland). The BCL-2/BCL-xL-specific BH3 mimetic ABT-737 and the Akt inhibitor MK-2206 were purchased from Selleckchem (Houston, TX). The MCL-1-specific BH3 mimetic A-1210477 and the BCL-xL-specific BH3 mimetic A-1331852 were purchased from Chemitek (Indianapolis, IN). The BCL-2/BCL-xL-specific BH3 mimetic navitoclax (ABT-263) was purchased from Adooq Bioscience (Irvine, CA). The pan-Caspase inhibitor Boc-D-FMK was purchased from BioVision (Milpitas, CA).

Propidium iodide and anti-β-actin (A1978) were purchased from Sigma (St Louis, MO, USA). Monoclonal antibodies against activated Bak (AM03) and phosphotyrosine (4G10) were purchased from Merck Millipore (Darmstadt, Germany). Monoclonal antibody against activated Bax (TACS-2281) was purchased from Trevigen (Gaithersburg, MD). The Mouse IgG APC-conjugated antibody was purchased from Bio-Techne R&D systems (Minneapolis, MN).

Monoclonal antibodies against JAK1 (CS-3344), JAK2 (CS-3230), phospho-Y1007/1008-JAK2 (CS-3776), JAK3 (CS-8827), phospho-Y980/981-JAK3 (CS-5031), non-phospho-T46-4EBP1 (CS-4923), phospho-T37/46-4EBP1 (CS-2855), phospho-S9-GSK3ß (CS-5558), phospho-T202/Y204-Erk (CS-9106), phospho-T389-p70S6K (CS-9234), phospho-T246-PRAS40 (CS-2997), MCL-1 (CS-5453), Pim-2 (CS-4730), phospho-Y694-STAT5 (CS-9359), phospho-Y705-STAT3 (CS-9145), eIF4E (CS-2067), eIF4G (CS-2469), c-Myc (CS-5605), and Bim (CS-2933) as well as polyclonal antibodies against phospho-S65-4EBP1 (CS-9451), phospho-S217/221-MEK (CS-9121), LC3B (CS-2775), phospho-T308-Akt (CS-9275), phospho-S473-Akt (CS-9271), phospho-Y1022/1023-JAK1 (CS-3331), and phospho-Y1054/1055-TYK2 (CS-9321) were purchased from Cell Signaling (Beverley, MA). Polyclonal antibodies against BCL-2 (SC-783), STAT5A (SC-1081), Bax (SC-493), Lyn (SC-15), JAK2 (SC-294), and c-Kit (SC-168) were purchased from Santa Cruz Biotechnology (Santa Cruz, CA). Antibodies against TYK2 (TL-20220) and BCL-xL (BD 610211) were purchased from BD Biosciences (San Jose, CA). Antibody against p62 was purchased from Medical and Biological Laboratories (Nagoya, Japan).

### Expression plasmids, transfection, and infection

Retrovirus vectors, pMIG/BCL-xL, pMIG/BCL-xL-mut1, and pMIG/BCL-xL-mut8, were gifts from Stanley Korsmeyer (Addgene plasmid #8790, 8791, 8792, respectively) [32], while pMIG was a gift from William Hahn (Addgene plasmid # 9044). A retroviral expression plasmid for a constitutively activated STAT5, pMXs-IG-STAT5A1^*^6, was constructed by subcloning the EcoRI/NotI fragment from pMXs-puro-STAT5A1^*^6 into the EcoRI/NotI site of the pMXs-IG vector, both of which were gifts from Toshio Kitamura. A retroviral expression plasmid for EGFP-LC3, pMXs-puro-EGFP-LC3, was constructed by subcloning the NheI (blunted)/SalI fragment from pEGFP-LC3, a gift from Karla Kirkegaard (Addgene plasmid # 11546), into the EcoRI (blunted)/XhoI site of the pMXs-puro vector, a gift from Toshio Kitamura.

To obtain PVTL-2 or HEL cells expressing the constitutively activated STAT5 mutant STAT5A1^*^6 (PVTL-2/pMXs-IG-STAT5A1^*^6 and HEL/pMXs-IG-STAT5A1^*^6), these cells were infected with the recombinant retrovirus obtained from PLAT-A cells transfected with pMXs-IG or pMXs-IG-STAT5A1^*^6, as described previously [50], and sorted for GFP expression by flow cytometry. To obtain HEL cells overexpressing BCL-xL or its mutants, HEL cells were infected with the recombinant retrovirus derived from pMIG, pMIG/BCL-xL, pMIG/BCL-xL-mut1, or pMIG/BCL-xL-mut8 and sorted for GFP expression by flow cytometry. PVTL-2 and HEL cells expressing EGFP-LC3 were obtained by infection of PVTL-2 or HEL cells with the recombinant retrovirus derived from pMXs-puro-EGFP-LC3, followed by selection with 1 μg/ml puromycin and sorting for GFP expression by flow cytometry.

### Analyses of cell proliferation and viability

Cell proliferation and viability were assessed by counting viable and nonviable cell numbers by the trypan blue-dye exclusion method. Cell viability was calculated by dividing number of viable cells by that of total cells. Viable cell numbers were also assessed using the Cell counting Kit-8 (Dojindo, Japan), according to the manufacturer's instructions. For combination studies, the synergy was assessed with the combination index (CI) of Chou and Talalay method using Compu Syn software [30]. The CI value less than 1 indicates synergism.

### Flow cytometric analyses for activation of Bax, Bak, and Caspase-3

Flow cytometric analyses for cell cycle and apoptosis, conformational changes of Bax and Bak, and cleavage of Caspase-3 were performed essentially as described previously [51, 52].

### Immunoblotting, immunoprecipitation, and cap-binding assays

Immunoblotting, immunoprecipitation, and cap-binding assays were performed essentially as described previously [52, 53], except for the following changes in lysis buffers. For immunoprecipitation of activated Bax, cells were lysed in a lysis buffer containing 1% CHAPS, 150 mM NaCl, 10 mM HEPES pH7.4, 1 mM phenylmethylsulfonyl fluoride and 10 μg/ml each of aprotinin and leupeptin. For immunoprecipitation of Bim, cells were lysed in a lysis buffer containing 1% Triton X-100, 20 mM Tris-HCl (pH 7.5), 137 mM NaCl, 1 mM EGTA, 50 mM NaF, 1.5 mM MgCl_2_, 1mM sodium orthovanadate, 10% glycerol, 1 mM phenylmethylsulfonyl fluoride and 10 μg/ml each of aprotinin and leupeptin.

### Confocal fluorescence microscopy

Cytospin samples of cells expressing EGFP-LC3B were prepared, and the slides were mounted with Prolong^®^ Gold antifade reagent with DAPI (Invitrogen, Carlsbad, CA). Images were acquired using the laser-scanning confocal microscope FluoView FV10i (Olympus, Tokyo, Japan) and analyzed with FV10 ASW10 software (Olympus).

## SUPPLEMENTARY MATERIALS FIGURES AND TABLES


