# Supplementary material for: Mechanisms for mTORC1 activation and synergistic induction of apoptosis by ruxolitinib and BH3 mimetics or autophagy inhibitors in JAK2-V617F-expressing leukemic cells including newly established PVTL-2
Source: Oncotarget. 2018 Jun 1;9(42):26834–51. doi: 10.18632/oncotarget.25515 (PMC6003557; doi:10.18632/oncotarget.25515)
Supplement: Supplementary file 1 [file oncotarget-09-26834-s001.pdf]

Mechanisms for mTORC1 activation and synergistic induction of apoptosis by ruxolitinib and BH3 mimetics or autophagy inhibitors in JAK2-V617F-expressing leukemic cells including newly established PVTL-2

SUPPLEMENTARY MATERIALS

Supplementary Table 1: Phenotypic differences of primary leukemic cells, PVTL-1, and PVTL-2

	Primary LC (%)	PVTL-1	PVTL-2
CD7	47.3	+	–
CD13	82.9	+	–
CD33	72.9	+	+
CD34	74.3	+	–
CD71	NT	NT	+
CD117	NT	+	+
HLA-DR	59.9	+	–

Abbreviations: LC, leukemic cells; NT, not tested.

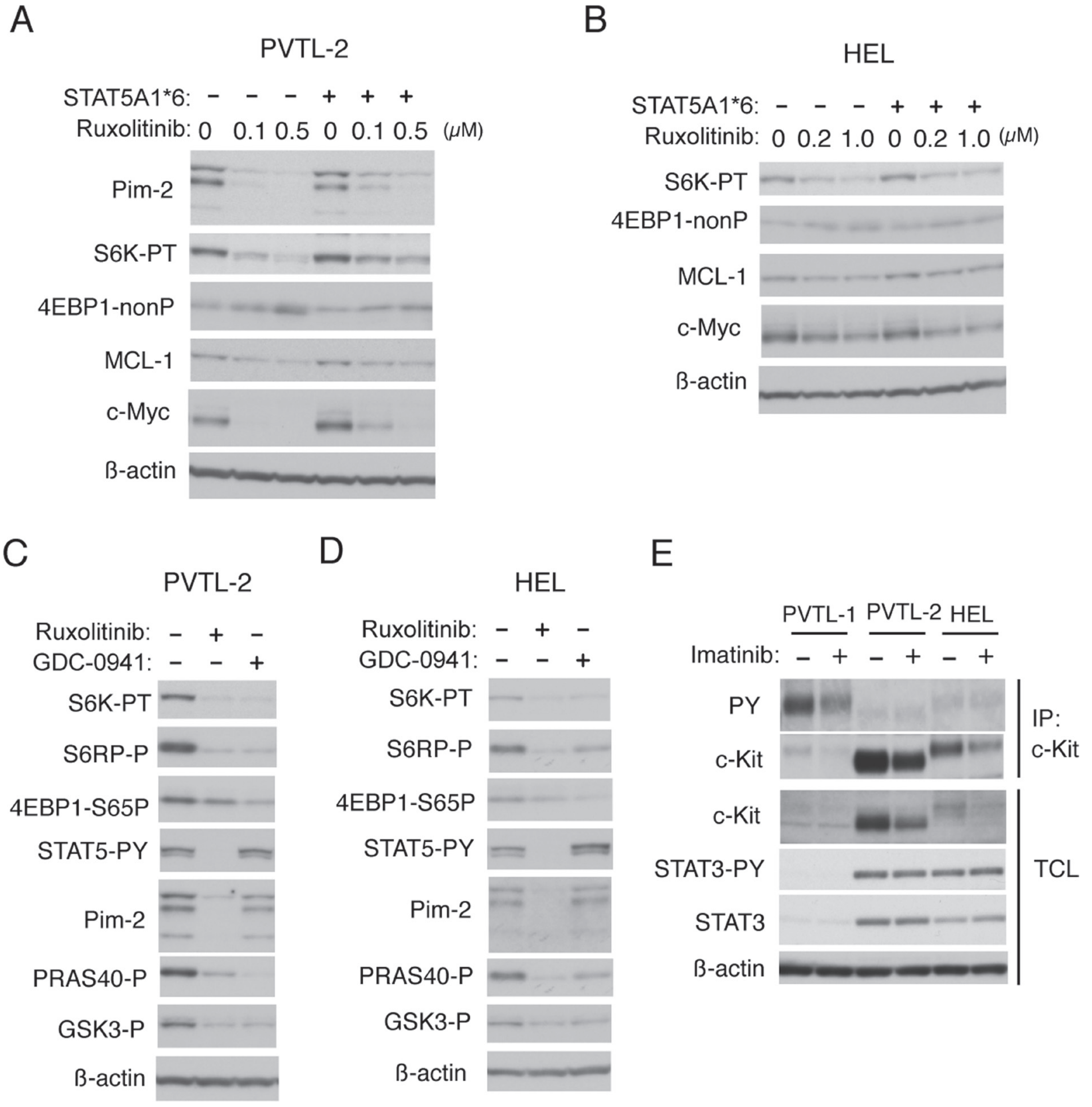

Supplementary Figure 1: Mechanisms of activation of the mTORC1 pathway by JAK2-V617F in PVTL-2 and HEL cells. (**A**, **B**) PVTL-2 or HEL cells transduced with STAT5A1*6 or vector control cells, as indicated, were treated with indicated concentrations of ruxolitinib for 6 h and subjected to immunoblot analysis. Abbreviations: S6K-PT, phospho-T389-p70S6 kinase; 4EBP1-nonP, non-phospho-T46-4EBP1. (**C**, **D**) PVTL-2 or HEL cells, as indicated, were treated for 6 h in ASF104 medium with 1 μM ruxolitinib or 1 μM GDC-0941, as indicated and analyzed. Abbreviations: S6RP-P, phospho-S240/244-S6RP; 4EBP1-S65P, phospho-S65-4EBP1; STAT5-PY, phospho-Y694-STAT5; PRAS40-P, phospho-T246-PRAS40; GSK3-P, phospho-S9-GSK3ß. (**E**) PVTL-1, PVTL-2, or HEL cells, as indicated, were treated for 6 h in ASF104 medium with or without 2 μM imatinib, as indicated, lysed, and immunoprecipitated with anti-c-Kit antibody. Immunoprecipitates (IP) and total cell lysates (TCL) were then subjected to immunoblot analyses with antibodies against indicated proteins. Abbreviations: PY, phosphotyrosine; STAT3-PY, phospho-Y705-STAT3.

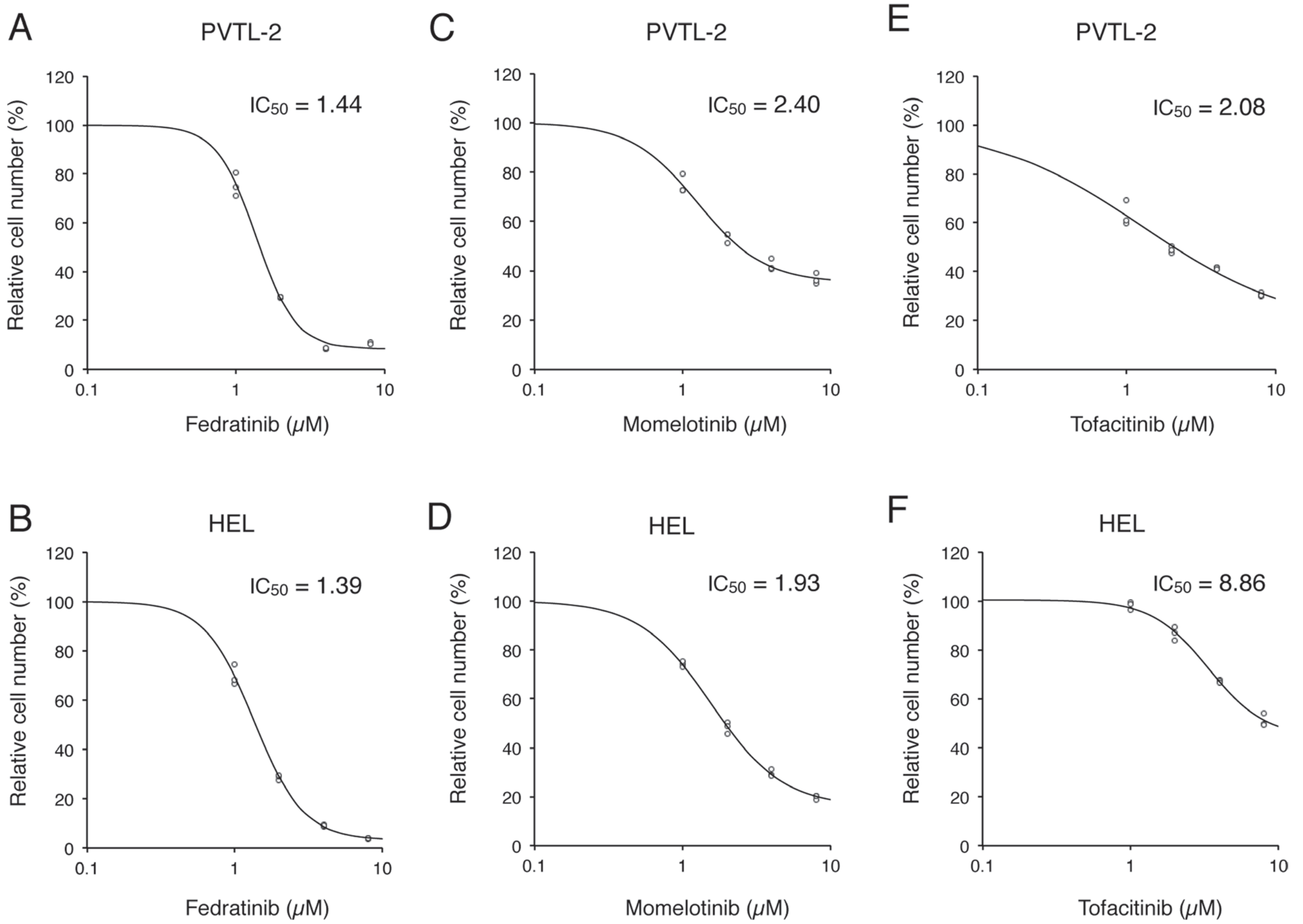

Supplementary Figure 2: Sensitivities of PVTL-2 and HEL cells to fedratinib, momelotinib, and tofacitinib. PVTL-2 cells (**A**, **C**, **E**) or HEL cells (**B**, **D**, **F**) were cultured with indicated concentrations of fedratinib (A, B), momelotinib (C, D), or tofacitinib (E, F) for 48 h. Viable cell numbers were measured by the Cell counting Kit-8. Relative cell numbers expressed as percentages of cell numbers without JAK inhibitors from triplicate samples are plotted with four-parameter logistic curves obtained by using ImageJ software with calculated IC_{50} (μM) indicated.

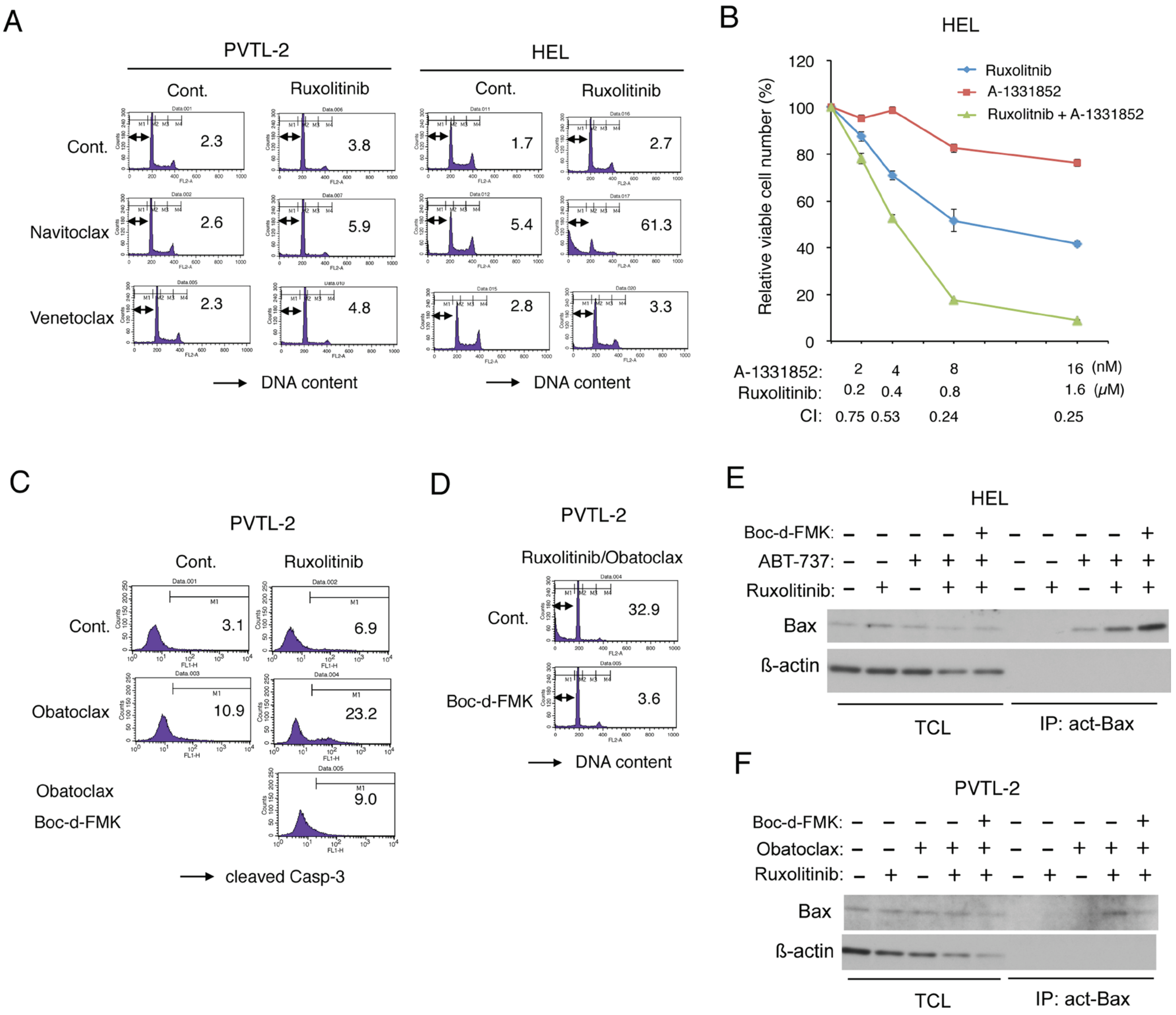

Supplementary Figure 3: Different sensitivities of PVTL-2 and HEL cells to various BH3 mimetics for caspase-dependent apoptosis involving activation of Bak and Bax. (**A**) PVTL-2 or HEL cells, as indicated, were cultured with 0.5 µM navitoclax or 2 µM venetoclax in the presence or absence of 1 µM ruxolitinib, as indicated, for 36 h and analyzed for the cellular DNA content by flow cytometry. Percentages of apoptotic cells with sub-G1 DNA content are indicated. (**B**) HEL cells were cultured with indicated concentrations of A-1331852 and ruxolitinib for 48 h. Viable cell numbers were measured in triplicate by the Cell counting Kit-8. Means of relative cell numbers expressed as percentages of cell numbers without inhibitors are plotted with error bars indicating standard errors. Combination index (CI) values obtained by the method of Chou and Talalay [50] are indicated. (**C**) PVTL-2 cells were cultured with 0.5 µM obatoclax and 100 µM Boc-d-FMK in the presence or absence of 1 µM ruxolitinib, as indicated, for 48 h and analyzed for activation of Caspase-3 by flow cytometry. Percentages of cells with cleaved Caspase-3 are indicated. (**D**) PVTL-2 cells were cultured with 1 µM ruxolitinib and 0.5 µM obatoclax in the presence or absence of 100 µM Boc-d-FMK, as indicated, for 48 h and analyzed for the cellular DNA content. (**E**) HEL cells were cultured with 1 µM ruxolitinib, 1 µM ABT-737, and 100 µM Boc-d-FMK, as indicated, for 24 h and analyzed. (**F**) PVTL-2 cells were cultured with 1 µM ruxolitinib, 0.5 µM obatoclax, and 100 µM Boc-d-FMK, as indicated, for 24 h and lysed. Total cell lysates (TCL) and immunoprecipitates (IP) obtained with antibody specific for the activated form of Bax (act-Bax) were subjected to immunoblot analysis using antibodies against indicated proteins.

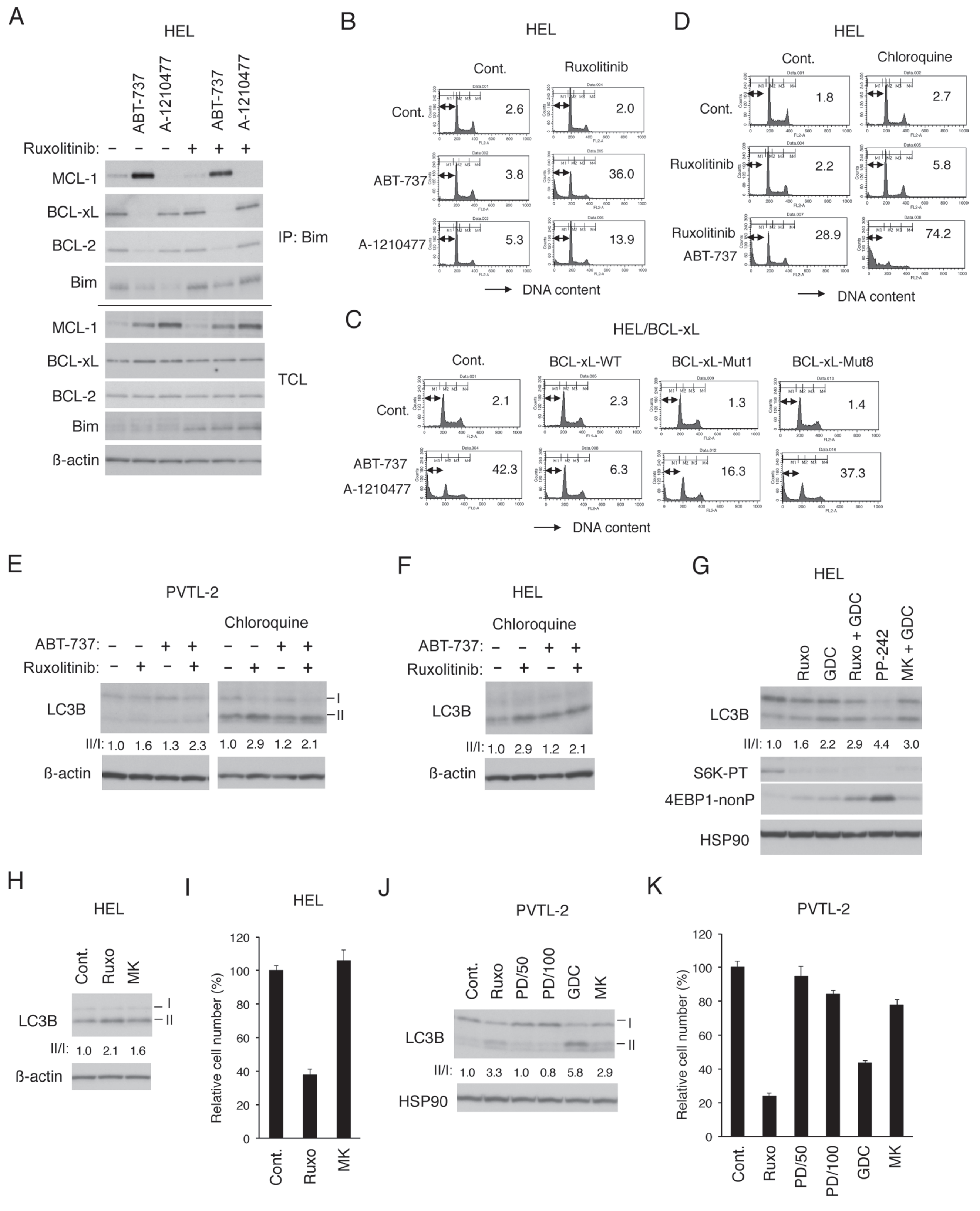

Supplementary Figure 4: Apoptosis induced in HEL cells by inhibition of anti-apoptotic BCL-2 family proteins and autophagy. (**A**) HEL cells were cultured with 1 µM ABT-737 or 10 µM A-1210477 in the presence or absence of 1 µM ruxolitinib, as indicated, for 8 h and lysed. Immunoprecipitates (IP) obtained with anti-Bim and total cell lysates (TCL) were subjected to immunoblot analysis using antibodies against indicated proteins. (**B**) HEL cells were cultured with 1 µM ABT-737, 10 µM A-1210477, and 1 µM ruxolitinib, as indicated, for 48 h and analyzed for the cellular DNA content by flow cytometry. Percentages of apoptotic cells with sub-G1

DNA content are indicated. (**C**) HEL cells transduced with the wild-type BCL-xL or its mutants (Mut1, Mut8) and vector control cells, as indicated, were left untreated as control or treated with the combination of 0.5 μM ABT-737 and 5 μM A-1210477 for 48 h and analyzed. (**D**) HEL cells were cultured with 1 μM ruxolitinib and/or 1 μM ABT-737 in the presence or absence of 100 μM chloroquine, as indicated, for 48 h and analyzed. (**E**) PVTL-2 cells were cultured with 1 μM ruxolitinib and/or 1 μM ABT-737 for 6 h with or without 40 μM chloroquine added, as indicated, for the last 4 h and subjected to immunoblot analysis using antibodies against indicated proteins. Positions of LC3B-I and LC3B-II as well as the relative ratio of LC3B-II/LC3B-I (II/I) determined by densitometric analysis are indicated. (**F**) HEL cells were cultured with 1 μM ruxolitinib and/or 1 μM ABT-737, as indicated, for 4 h in the presence of 40 μM chloroquine and analyzed. (**G**) HEL cells were treated for 6 h with 1 μM of ruxolitinib (Ruxo), GDC-0941 (GDC), PP242, or MK-2206 (MK), as indicated, and analyzed. Abbreviations: S6K-PT, phospho-T389-p70S6 kinase; 4EBP1-nonP, non-phospho-T46-4EBP1. (**H**, **I**) HEL cells were cultured with 1 μM ruxolitinib (Ruxo) or 1 μM MK-2206 (MK), as indicated, for 4 h with 40 μM chloroquine added for the last 2 h for immunoblot analysis or cultured for 48 h for cell proliferation assay in triplicate. Means of relative cell numbers are plotted with error bars indicating standard errors. (**J**, **K**) PVTL-2 cells were cultured with 1 μM ruxolitinib (Ruxo), 50 μM PD98059 (PD/50), 100 μM PD98059 (PD/100), 1 μM GDC-0941 (GDC), or 1 μM MK-2206 (MK), as indicated, for 6 h for immunoblot analysis or for 48 h for cell proliferation assay.
